# Programmable Hydration Pathways Enable Reconfigurable Ionic Thermoelectrics for Energy Harvesting and Thermal‐Tactile Interaction

**DOI:** 10.1002/advs.75158

**Published:** 2026-04-07

**Authors:** Zehao Zhao, Yun Shen, Dongyan Xu

**Affiliations:** ^1^ Department of Mechanical and Automation Engineering The Chinese University of Hong Kong Hong Kong China

**Keywords:** energy harvesting, hydration pathways, ionic thermoelectrics, proton thermodiffusion, thermal‐tactile interaction

## Abstract

Ionic thermoelectric (iTE) materials deliver large thermovoltages but face a trade‐off among thermopower, speed, and stability. Here we realize reconfigurable iTE performance in polyquaternium hydrogels by programming hydration pathways across scales, coupling microscopic solvation and polymer‐ion interactions to mesoscopic water channels and macroscopic boundary conditions. This hydration‐gated protonics framework decouples ion transport barriers from water activity and thermal gradient, enabling two distinct operating states. An open, breathable state amplifies asymmetric water activity and yields ultrahigh thermopower of 44.8 mV K^−^^1^ in PAETC/PSS hydrogels. A sealed state suppresses hydration exchange, yields subsecond dynamics, and supports long‐term stability. Guided by thermo‐hydration co‐design that treats the hydration boundary as a tunable parameter, we translate the mechanism into two platforms. A wearable energy‐harvesting module with a stable vertical bias ΔT = 3.0 K delivers a thermovoltage of ∼0.6 V, while a high‐sensitivity, fast‐response (0.5 s), long‐term‐stable (> 90 days) iTE sensor array with sealed‐mode reliability is integrated with a robotic hand for thermal–tactile interaction.

## Graphical abstract

Programmable hydration pathways enable reconfigurable ionic thermoelectrics in polyquaternium hydrogels. By coupling microscopic solvation, mesoscale water channels, and macroscopic boundary control, hydration‐gated protonics decouples thermopower, response speed, and stability. The system delivers ultrahigh thermopower (44.8 mV K^−^^1^) in open mode and rapid, stable sensing in sealed mode, enabling both low‐grade heat harvesting and thermal‐tactile human–machine interaction.

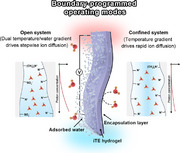

## 1 Introduction

Ionic thermoelectric (iTE) materials are promising for low‐grade heat harvesting and high‐sensitivity thermal sensing, owing to their remarkably high thermopower (*S*_i_) and inherently low thermal conductivity. The thermal‐to‐electrical energy conversion in iTE materials is governed by the Soret or thermodiffusion effect [1, 2]. In ionic conductors, the thermodiffusion process involves the directed migration of both cations and anions from the high temperature region to the low temperature region. In non‐equilibrium systems, the difference in heats of transport ($Q_{+}^{*}\;\neq \;Q_{-}^{*}$) by ions leads to distinct concentration gradients for cations and anions, generating a measurable thermovoltage [3]. Therefore, maximizing the *S*_i_ critically depends on the disparity in thermophoretic mobility between cations and anions.

Recent studies have revealed that ion diffusion in solid‐state polymeric frameworks proceeds through an activated hopping mechanism between adjacent sites, where $Q_{\pm }^{*}$ is governed by the activation enthalpy (Δ*H*_±_) of charge carriers [3]. Various strategies have been developed to modulate Δ*H*_±_, including ion selection (e.g., H^+^/Cl^−^ [4], Na^+^/[TFSI]^−^ [5], [EMIM]^+^/[DCA]^−^) [6], polymer network modifications [7, 8], and electrode engineering [9]. Most of these approaches are highly sensitive to relative humidity (RH), which is linked to companion field arising from nonuniform temperature distributions [10]. Specifically, this companion field arises from asymmetric water activity (*a*_w_) under a thermal gradient​, where *a*_w_ is defined as the ratio of the equilibrium vapor pressure of water within a material to that of pure water at the same temperature. At vapor equilibrium, *a*_w_ equals the equilibrium RH expressed as a fraction, providing a quantitative link between ambient humidity and the internal hydration state. Water naturally migrates from regions of higher *a*_w_ ​to lower *a*_w_​; thus, temperature gradients can induce corresponding *a*_w_ ​gradients through evaporation and condensation, leading to persistent hydration asymmetry during operation. This asymmetry provides a tunable handle on ion solvation, dissociation, and effective transport barriers.

At the microscopic and mesoscopic scales, hydration governs both carrier density and the topology of transport pathways. For classical hydrated ionomers such as Nafion, proton conductivity rises with water uptake, as enhanced ion dissociation and the formation of percolated aqueous channels with nanometer‐scale diameters and connected hydrogen‐bond networks facilitate efficient charge transport. Proton transport proceeds through a balance between the vehicle mechanism, as migration of hydrated ions, and Grotthuss‐type structural diffusion along hydrogen‐bonded chains [11]. In addition, water‐polymer interactions, e.g., hydrophilic –(CH_3_)_3_N^+^/–SO_3_^−^/–OH groups and hydrophobic alkyl chains, may drive microphase separation, leading to percolated ionic networks that facilitate long‐range transport when optimally scaled [12, 13, 14]. These relationships extend to polyelectrolyte hydrogels, where confined water, polymer–ion interactions, and mesoscale phase morphology collectively determine the activation barriers for ion transport. This framework also offers a route to disentangle the inherent trade‐off among thermopower, response speed, and operational stability. By treating hydration not as a fixed environmental constraint but as a programmable variable, ion‐transport barriers can be decoupled from *a*_w_ and thermal gradients. In this view, boundaries function not as passive enclosures but as active design knobs to regulate water exchange, companion fields, and effective driving forces, thereby enabling programmable hydration pathways within a single polymer platform.

**FIGURE 1:**
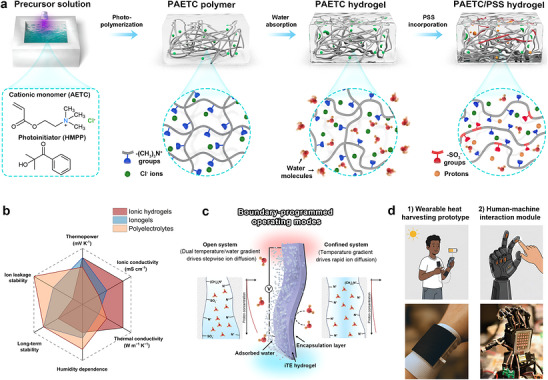
(a) Schematic illustration of the synthesis of PAETC polymer, formation of PAETC and PAETC/PSS hydrogels, and their molecular structures. (b) Comparison of different ionic conductors. (c) Boundary‐programmed operating modes: open state and sealed state. (d) Schematic illustration and photographs of two application modules utilizing PAETC‐based iTE hydrogels for low‐grade heat harvesting and thermal sensing.

In this work, we introduce hydration‐gated protonics (HGP), a design concept that programs hydration pathways across multiple scales through co‐regulation of composition, microstructure, and boundary conditions. Specifically, we strategically designed polyquaternium poly[2‐(acryloyloxy)ethyl]trimethylammonium chloride (PAETC) hydrogels and their composites with polystyrene sulfonic acid (PSS) via mild free‐radical polymerization (Figure 1a). PAETC is a typical polyelectrolyte comprising hydrophilic quaternary‐ammonium (−(CH_3_)_3_N^+^) domains and hydrophobic alkyl chains, which enables spontaneous proton (H^+^) release while confining counterions (Cl^−^) under low water conditions. This addresses longstanding issues in conventional ionic conductors, including ion leakage and poor performance at low liquid content (Figure 1b). In the sealed state, the encapsulation layer suppresses evaporation, allowing the temperature gradient to dominate the driving force. Consequently, liberated protons rapidly migrate from the hot end to the cold end, yielding a fast response and a drift‐free output. In the open state, the exposed surface establishes a sustained *a*_w_ gradient that works synergistically with the thermal gradient (Figure 1c). The resulting hydration asymmetry prolongs proton migration and delivers a high *S*_i_ of 36.6 mV K^−^^1^ in pristine PAETC. Incorporating PSS creates a proton‐rich and scale‐optimized hydration landscape that homogenizes water pathways and lowers ion‐transport barriers, further boosting *S*_i_ to 44.8 mV K^−^^1^ under open operation. Taken together, the HGP strategy enables dual‐mode operation in PAETC‐based iTE hydrogels: an open, high‐thermopower regime for low‐grade heat harvesting, and a sealed, rapid‐response regime for robust thermal perception. This reconfigurable behavior is further demonstrated in two application modules, including a wearable heat‐harvesting patch that maintains a stable vertical hydro‐thermal gradient, and a thermal‐tactile human‐machine interface capable of temperature mapping, trajectory tracking, and robotic gesture control (Figure 1d).

## 2 Results and Discussion

### 2.1 Mechanical and iTE Properties of Heterogenous PAETC Hydrogel

**FIGURE 2:**
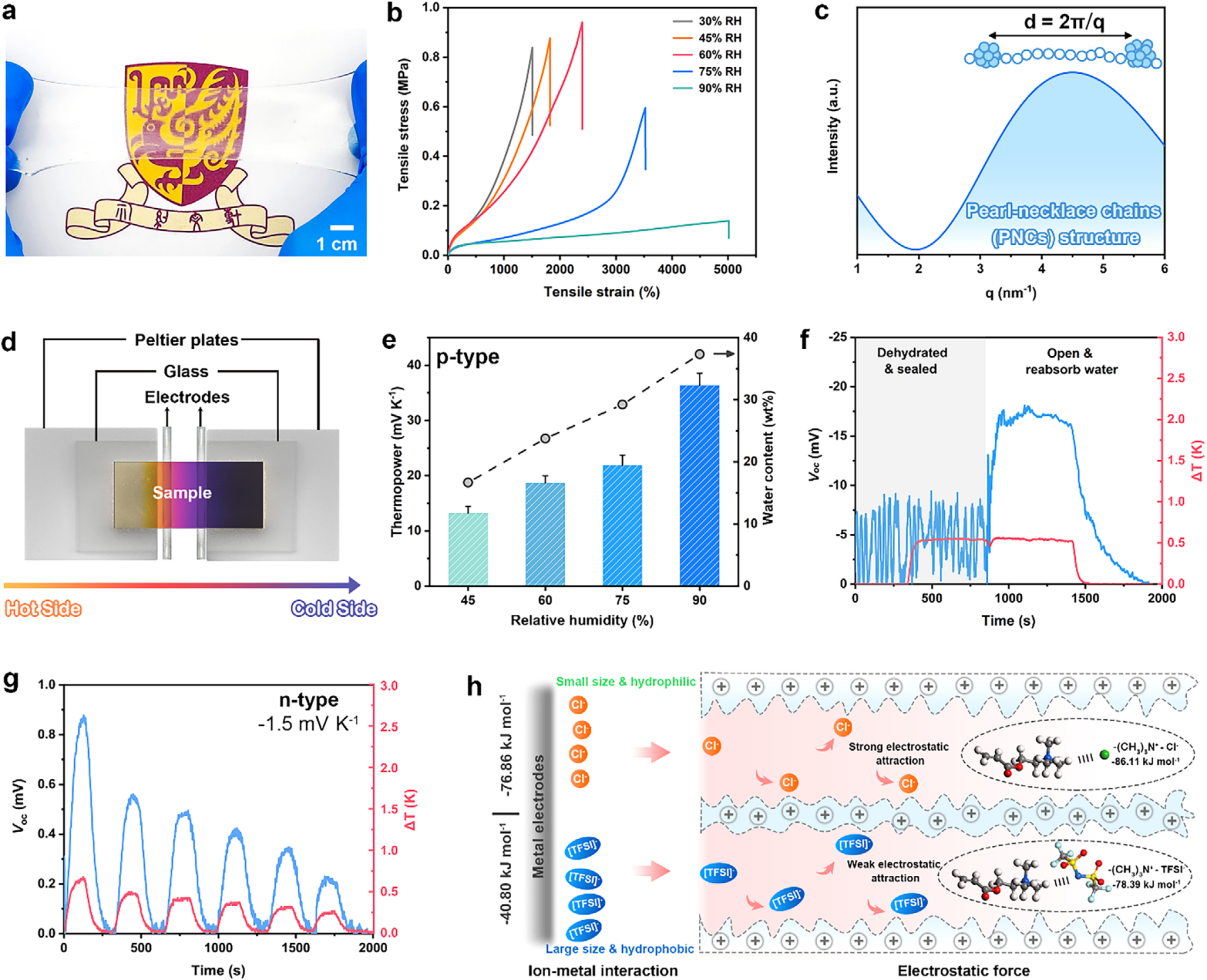
(a) Photograph of a representative PAETC hydrogel. (b) Tensile stress–strain curves of PAETC hydrogels equilibrated at different RH levels. (c) 1D SAXS intensity profile of PAETC hydrogel. (d) Schematic representation of the test setup and simulated temperature distribution across the sample. (e) Dependence of thermopower and water content of PAETC hydrogels on RH. (f) *V*_oc_ and Δ*T* profiles of dehydrated PAETC polymer under sealed and open conditions. (g) *V*_oc_ and Δ*T* profiles of P[AET][TFSI] polymer. (h) Schematic illustration of electrostatic interactions between polymer chains and Cl^−^/[TFSI]^−^ ions, and ion‐metal interfacial interactions.

Owing to the hydrophilic −(CH_3_)_3_N^+^ groups, PAETC readily absorbs ambient moisture, forming hydrogels whose water content correlates directly with RH. Quantitative analysis reveals that the water content of PAETC hydrogels increases progressively with ambient RH, reaching approximately 9.8 wt.%‐37.3 wt.% in the range of 15%‐90% RH. In the absence of absorbed water, PAETC polymer displays a brittle, plastic‐like nature with minimal elongation. By contrast, PAETC hydrogels exhibit high optical transparency, superior tensile properties, and rapid mechanical recovery (Figure 2a; Figure S1). The mechanical response of PAETC hydrogels was systematically investigated after equilibration at various RH levels, revealing a pronounced dependence on water uptake. At low humidity, restricted water absorption enhances polymer chain mobility, resulting in tough fracture behavior marked by elastic deformation, yielding, strain softening, and subsequent stress hardening (Figure S2). At 15% RH, the hydrogel demonstrates a tensile strength of 4.54 MPa and an elongation at break of 354%. With increasing RH, water uptake further lowers the energy barriers for polymer chain displacement. In the 30–90% RH range, PAETC hydrogels exhibit exceptional stretchability, achieving maximum elongation exceeding 5000% with a peak tensile strength of 0.94 MPa (Figure 2b). Repeated cyclic stress–strain testing and rheological measurements corroborate the outstanding elasticity of PAETC hydrogels (Figure S3).

The mechanical properties of hydrogels are determined by the architecture of the polymer network and the nature of inter‐ and intramolecular interactions. The pronounced hyperelasticity of PAETC hydrogels is attributed to the formation of reversible pearl‐necklace chains (PNCs) through heterogeneous hydration under water‐deficient conditions (Figure S4). This distinctive structure arises from the interplay between hydrophobic interactions of alkyl chains and electrostatic interactions of ionic groups [15]. Evidence for the PNCs structure is provided by 1D small‐angle X‐ray scattering (SAXS) profile, with a broad peak centered at 4.5 nm^−^^1^ corresponding to a bead‐to‐bead spacing of ∼1.39 nm (Figure 2c).

The iTE performance of PAETC hydrogels was systematically evaluated across a range of RH (45%, 60%, 75%, and 90%) using a planar setup (Figure 2d). As shown in Figure 2e and Figure S5, the hydrogels exhibit a positive *S*_i_, comparable to that of PDDA [16], with *S*_i_ displaying a strong positive correlation with increasing RH. Notably, the maximum *S*_i_ reaches 36.6 mV K^−^^1^ at 90% RH. This enhancement in thermopower is accompanied by a marked RH‐dependent increase in ionic conductivity (*σ*_i_), which increases from 0.5 to 10.4 mS cm^−^^1^ as RH rises (Figure S6). To investigate the intrinsic iTE properties of PAETC polymer in the absence of water, we performed thermovoltage measurements on fully dehydrated samples. Dehydrated PAETC exhibited no detectable response under a thermal gradient, indicating negligible thermophoretic mobility of Cl^−^ ions (Figure 2f). This strong suppression of ion transport is likely a consequence of interfacial ion‐metal interactions at the electrode and electrostatic binding of anions to the polymer backbone.

To examine electrode effects, we compared the thermovoltage output of both PAETC polymer and hydrogel on Ag, Cu, and Au electrodes. As shown in Figure S7, PAETC polymer demonstrates open‐circuit behavior on Ag and Cu electrodes, but a weak n‐type response on Au. This suggests pronounced adsorption and immobilization of Cl^−^ ions on the Ag/Cu surface, whereas Au exhibits weaker ion affinity. Conversely, PAETC hydrogel shows a robust p‐type thermopower on Ag (25.12 mV K^−^^1^) and Cu (23.84 mV K^−^^1^) electrodes, but displays a small, rapidly decaying voltage on Au. This transient response is attributed to the rapid migration of mobile Cl^−^ ions, which neutralize the thermovoltage arising from cation accumulation at the cold end [17, 18]. Considering that Cu is easily oxidized, Ag electrodes are selected for thermopower measurements in this work. In addition, to exclude the possibility of metal‐involving redox reactions at the Ag electrode, cyclic voltammetry was performed in a symmetric Ag | gel | Ag configuration. No pronounced paired anodic/cathodic peaks were observed within the relevant voltage window (Figure S8). To further probe the influence of anion‐polymer and anion‐electrode interactions, we implemented an anion exchange strategy by replacing Cl^−^ with hydrophobic bis(trifluoromethanesulfonyl)imide ([TFSI]^−^) ions (Figure S9). Owing to their larger size and weaker binding, [TFSI]^−^ ions interact less strongly with the polymer matrix and electrode surfaces, thereby enabling efficient thermodiffusion. As a result, P[AET][TFSI] demonstrates a stable n‐type thermopower (−1.5 mV K^−^^1^) on Ag electrodes (Figure 2g). Molecular dynamics (MD) simulations further reveal that [TFSI]^−^ ions exhibit significantly lower binding energies with –(CH_3_)_3_N^+^ groups and electrodes than Cl^−^ ions (Figure 2h). These results highlight the limited mobility of Cl^−^ ions in PAETC polymer and hydrogels [19].

### 2.2 Hydration‐Induced Thermovoltage Generation Mechanisms

**FIGURE 3:**
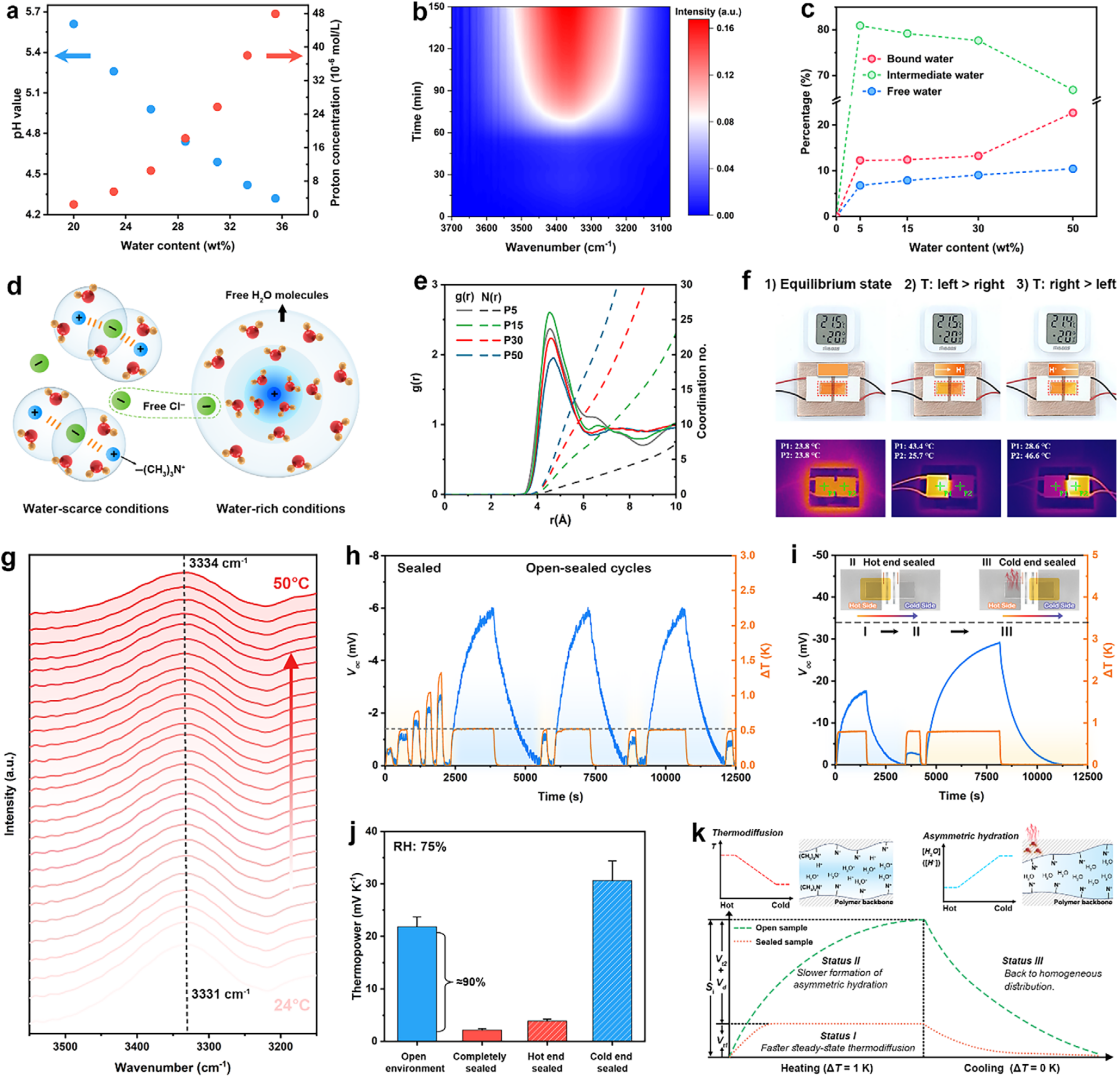
(a) pH and [H^+^] of monomer solutions at different water contents. (b) In situ FTIR hydration dynamics at 40% RH over 150 min. (c) Percentages of water states (bound, intermediate, and free) extracted through fitting FTIR spectra for PAETC hydrogels with 5, 15, 30, and 50 wt.% water contents. (d) Solvation structures under water‐scarce versus water‐rich conditions. (e) RDF and coordination number of water molecules in the first hydration shell for P5, P15, P30, and P50 samples. (f) Visualization of proton distribution under a temperature gradient by MR. (g) In situ FTIR spectra of PAETC hydrogels measured across a temperature range of 24–50(C. (h) *V*_oc_ and Δ*T* profiles recorded under sealed conditions and during repeated open‐sealed cycles. (i) Boundary programming with three sealing configurations under identical Δ*T*: I fully open, II hot end sealed, III cold end sealed. (j) *S*_i_ of PAETC hydrogels under different sealing conditions. (k) Schematic illustration of the synergistic thermovoltage generation mechanisms based on thermodiffusion and asymmetric hydration in PAETC hydrogels.

We next examined the role of water in the p‐type performance of PAETC hydrogels and sought to identify the dominant cation species contributing to thermovoltage generation. To assess intrinsic proton production of PAETC hydrogels in the presence of water, we measured the pH of AETC precursor solutions across a range of water contents (Figure 3a; Figure S10). The commercial AETC solution (20 wt.% in water) had an initial pH of 5.61, which progressively decreased to 3.28 upon increasing the water content to 80 wt.%, confirming proton generation in the solution [20, 21]. According to the Stokes–Einstein equation, protons possess the highest mass diffusion coefficient in aqueous environments (9.311 × 10^−^^5^ cm^2^ s^−^^1^), attributed to their small ion radius [22, 23]. In PAETC hydrogels, proton production primarily originates from solvation interactions between the −(CH_3_)_3_N^+^ groups and the first hydration shell of surrounding water molecules [24]. Specifically, the hydroxyl groups of water establish hydrogen bonds with unsaturated −(CH_3_)_3_N^+^ groups (N^+^···H─O), which facilitates proton release.

The dynamic water states within PAETC hydrogels were investigated using in situ Fourier‐transform infrared (FTIR) and attenuated total reflectance (ATR)‐FTIR spectroscopy. The O─H stretching vibration band increased in intensity as dehydrated PAETC equilibrated at 40% RH over 150 min, indicating progressive hydration (Figure 3b). Synchronous and asynchronous 2D correlation ATR‐FTIR analyses (Figure S11) further delineated three distinct water states within the PNCs structure: bound, intermediate, and free water [25]. Deconvolution of ATR‐FTIR spectra for PAETC hydrogels containing 5, 15, 30, and 50 wt.% water (P5, P15, P30, and P50) over the 3100–3800 cm^−^^1^ range revealed that intermediate water is predominant in all samples, while the fractions of bound and free water systematically increase with higher water contents (Figure 3c; Figure S12). Generally, water molecules are predominantly bonded to cations. Under water‐scarce conditions, an insufficient number of water molecules cannot fully screen the cationic charges, forcing anions into the solvation shell. In water‐rich conditions, ions are fully dissociated and can form solvation structures composed solely of water molecules (Figure 3d). MD simulations provide further insight on the structure of the first hydration shell. The radial distribution function (RDF) analysis shows that the first hydration shell forms at 4.6 Å, and the coordination number of water molecules within this shell rises with increasing water content (Figure 3e). Enhanced coordination strengthens interactions between water molecules and −(CH_3_)_3_N^+^ groups, thereby promoting proton generation. Collectively, these results establish a direct correlation among water content, hydration state, and proton activity in PAETC hydrogels [26, 27].

Other protic solvents capable of proton dissociation may, in principle, also induce p‐type thermoelectric behavior. To verify this, we partially substituted water in PAETC hydrogels with either aprotic (e.g., ethyl acetate) or protic (e.g., methanol, isopropyl alcohol) solvents and assessed their impact on *S*_i_. As shown in Figure S13, solvent‐substituted samples exhibited a marked reduction in *S*_i_ relative to the water‐based hydrogels, with ethyl acetate, which cannot support proton dissociation, producing the lowest value. For the protic solvents, *S*_i_ scaled with the ratio of their dissociation constant to dielectric constant (*K*_d_/*ε*), in agreement with previous studies [28]. These findings highlight the efficient proton generation within the first solvation shell of protic‐solvent‐based PAETC hydrogels.

To directly visualize the proton gradient formed under a thermal gradient, methyl red (MR) was introduced as an acid‐base indicator. MR transitions from red to yellow within a pH range of 4.4‐6.2. When a light red MR‐containing sample was symmetrically placed on two Peltier modules and heated asymmetrically (Figure 3f), the warmer side turned yellow while the colder side deepened to red, indicating a lower pH at the cold end [29]. This visual observation provides direct evidence for asymmetric proton distribution under a temperature gradient in PAETC hydrogels.

Proton redistribution tightly couples to water content and hydration state, remaining in dynamic equilibrium under varying humidity and temperature. In situ ATR‐FTIR spectra of the PAETC hydrogel heated from 24°C to 50°C (Figure 3g) show a gradual decline in peak intensity and a blue shift of vibrational bands, evidencing a loss of total water content and a reduced proportion of bound water at higher temperatures. This behavior stems from the negative Δ*H*_hyd_ and the consequent weakening of hydrogen bonding, which modulates the proton dissociation constant (*K*_d_​). Within the HGP framework, these temperature‐driven changes represent programmed hydration pathways governed by the water activity profile *a*_w_, thereby reshaping both dissociation and transport energetics. As a result, the output voltage of the PAETC hydrogel arises from both the Soret effect (*V*_t_​) and asymmetric proton dissociation across the temperature gradient (*V*_d_). According to the Nernst equation, this dissociation‐induced potential is given by

(1) $$E=E^{0}-\frac{RT}{F}\ln\left(\frac{{\left[H^{+}\right]}_{\mathrm{hot}}}{{\left[H^{+}\right]}_{\mathrm{cold}}}\right)=E^{0}-\frac{RT}{F}\ln\left(\frac{K_{d,\mathrm{hot}}}{K_{d,\mathrm{cold}}}\right)$$

where *E* ^0^ is the standard cell potential (zero for non‐Faradaic concentration cell), *R* is the gas constant, *T* is the absolute temperature, *F* is the Faraday constant, [H^+^] is the proton concentration.

To elucidate the effect of hydration dynamics on iTE performance, we measured the thermovoltage of PAETC hydrogels under sealed conditions designed to minimize variations in hydration structure, including water evaporation and bound water dynamics. Under equilibrium, the relationship between the Gibbs free energy change of dissociation (Δ*G*_d_) and *K*_d_ is expressed as

(2) $$\Delta \;G_{d}=\Delta H_{d}-T\Delta \;S_{d}=-RT\ln K_{d}$$

where Δ*H*_d_ and Δ*S*_d_ represent the enthalpy and entropy changes of dissociation, respectively. Applying the Van't Hoff equation for small temperature differences (Δ*T* ≪ *T*), *V*_d_ can be linearized as

(3) $$V_{d}=-\frac{RT}{F}\ln\;\left(\frac{K_{d,\;\mathrm{hot}}}{K_{d,\;\mathrm{cold}}}\right)=-\frac{\Delta H_{d}}{F}\cdot \frac{\Delta T}{T}$$

This derivation shows that *V*_d_ partially offsets the thermodiffusion‐driven potential (Δ*H*_d_​ > 0 for endothermic dissociation) due to their opposite polarities. Under small temperature gradients, however, this counteracting effect is negligible. Consequently, *S*_i_ is primarily governed by steady‐state proton thermodiffusion and is therefore markedly suppressed in sealed systems (Figure 3h). In contrast, under typical open conditions, the hydration structure evolves asymmetrically in response to the temperature gradient, leading to higher thermovoltage output and longer response times. HGP treats the boundary as a tunable parameter that establishes a sustained ∇*a*_w_ through controlled evaporation–condensation, which enhances proton dissociation at the cold end and introduces a hydration‐assisted diffusion component. Importantly, the output can be modulated by selectively adjusting the degree of hydration asymmetry between the hot and cold ends. As shown in Figure 3i, three distinct thermovoltage profiles emerge under identical thermal gradients but different sealing configurations. In stage I (fully open), the system exhibits baseline performance governed by natural hydration asymmetry. In stage II (hot end sealed), suppression of hydration asymmetry leads to an 82.3% reduction in thermovoltage and a much faster response. Conversely, in stage III (cold end sealed), enhanced hydration asymmetry slows the approach to steady state and increases the thermovoltage by 40.3%.

Figure 3j summarizes the *S*_i_ of PAETC hydrogels at 75% RH under different sealing conditions. Obviously, PAETC hydrogels achieve a high *S*_i_ under open conditions, and rapid, stable output under sealed conditions. The underlying mechanism based on thermodiffusion and asymmetric hydration is illustrated schematically in Figure 3k. In detail, the *S*_i_ of the fully sealed sample is approximately 10% of that in the open system, corresponding to the net steady‐state proton thermodiffusion (*V*_t1_). The remaining 90% of the thermopower observed in the open system arises from two additional contributions: i) asymmetric proton dissociation (*V*_d_) and ii) hydration‐enhanced proton diffusion (*V*_t2_​). Asymmetric proton dissociation (*V*_d_) is governed by *K*_d_, which is sensitive to temperature, water content, and the fraction of bound water. At the cold end, higher water content and greater hydration reduce the endothermic enthalpy barrier (Δ*H*_d_​) and increase the entropy gain (Δ*S*_d_), resulting in a more negative Gibbs free energy change (Δ*G*_d_ < 0). This shift promotes spontaneous proton dissociation at the cold end and enhances output voltage, as further supported by the drip experiment (Figure S14). The increase in proton diffusion (*V*_t2_​) can be explained by the ion hopping dynamics model [3], where ion transport occurs via thermally activated jumps between adjacent sites. In the open state, the concurrent water‐concentration gradient lowers the hopping barrier toward the cold end and increases pathway connectivity, which accelerates directional transport. In the sealed state, the barrier remains high, and the pathway is hydration‐static, which explains the faster but smaller steady output [30, 31]. Upon removal of the temperature gradient, proton and water distributions kinetically return to a homogeneous equilibrium state.

### 2.3 Microscopic Hydration Engineering via PSS Incorporation

The macroscopic water concentration gradient resulting from asymmetric hydration markedly enhances proton transport in PAETC hydrogels. However, the influence of nanoscale hydration heterogeneity, particularly the phase‐separated PNCs structure, remains to be fully elucidated. Within the PNCs structure, discontinuous liquid‐phase domains can create tortuous migration pathways, increasing the energy barriers for proton diffusion. To improve proton transport, PSS was incorporated into the PAETC matrix at varying concentrations (5‐20 wt.%) to form PAETC/PSS (PAS) composite hydrogels. PSS can enhance proton migration via three primary mechanisms: 1) increasing local proton concentration through dissociation of sulfonic acid groups (pKa = 2.0), 2) programming pathway topology by homogenizing hydration structures by electrostatic complexation, and 3) coupling to macroscopic *a*_w_ control by dynamically regulating water gradients [32, 33, 34, 35].

**FIGURE 4:**
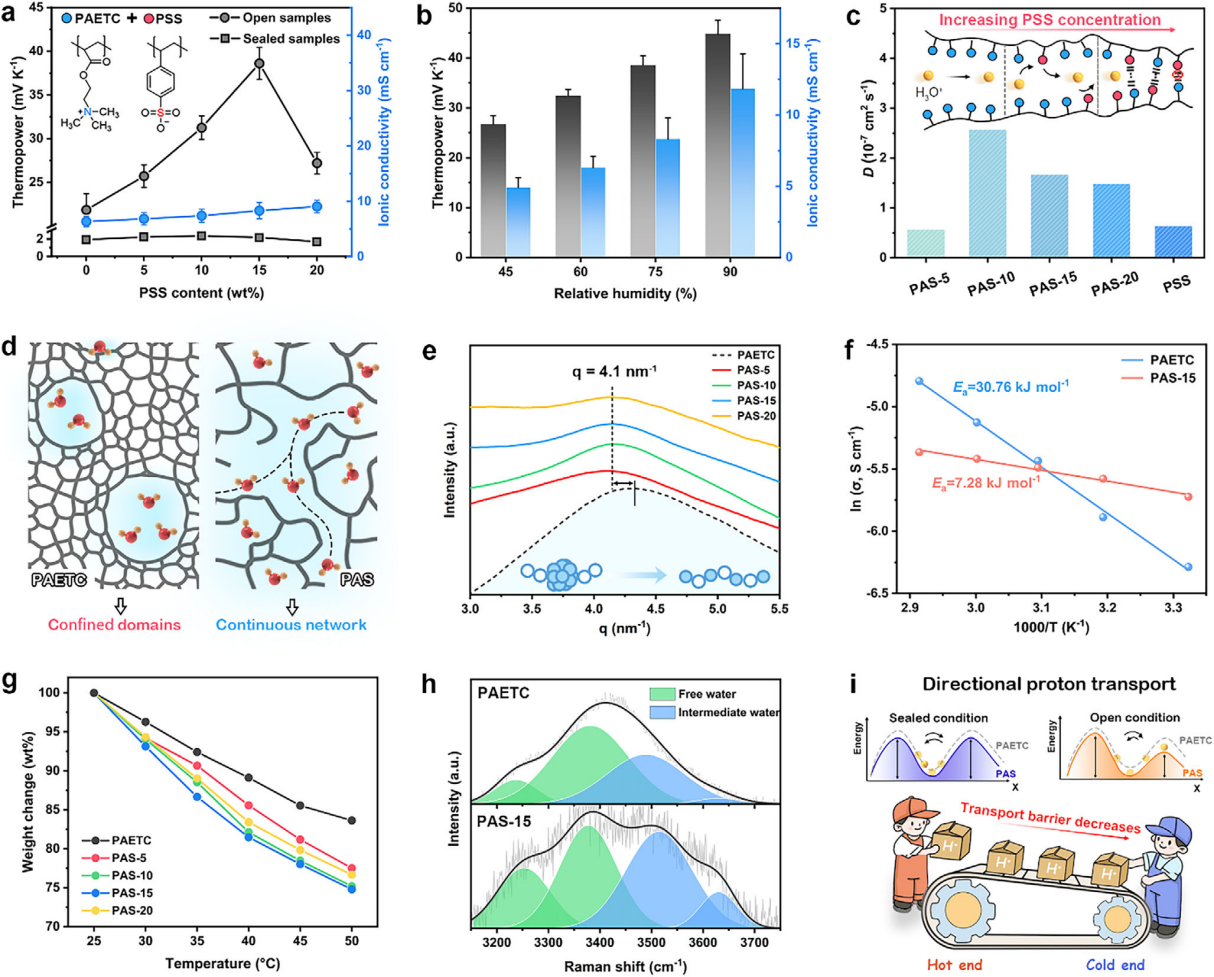
(a) *S*_i_ and *σ*_i_ of PAS hydrogels as a function of PSS content. Inset: molecular structures of PAETC and PSS. (b) *S*_i_ and *σ*_i_ of PAS‐15 hydrogels at different RH levels. (c) H_3_O^+^ diffusion coefficients derived from MSD analysis in MD simulations. Inset: schematic illustration of ion nanochannels. (d) Schematic illustration of water distribution in PAETC and PAS hydrogels. (e) 1D SAXS intensity profiles demonstrating structural evolution with increasing PSS content. (f) Ion migration energy barriers of PAETC and PAS‐15 hydrogels extracted by linear fitting of ln(*σ*_i_)‐1000/*T* data. (g) Temperature dependence of weight change of PAETC and PAS hydrogels, reflecting water loss. (h) Deconvoluted Raman spectra of PAETC and PAS‐15 hydrogels, showing the distribution of water states. (i) Schematic representation of directional proton transport in PAS hydrogels.

The *S*_i_ and *σ*_i_ of PAS hydrogels with different PSS contents and at various RH levels are shown in Figure 4a,b, respectively. Similar to PAETC hydrogels, the iTE performance of PAS hydrogels was assessed under both open and sealed conditions (Figures S15 and S16). Under sealed conditions, moderate PSS incorporation (≤ 15 wt.%) led to a slight increase in *S*_i_, whereas higher PSS content (20 wt.%) inhibited proton diffusion. In open environments, where boundaries maintain ∇*a*_w_ > 0, composition and boundary co‐program iTE performance of PAS hydrogels. At 75% RH, *S*_i_ increased with PSS content, peaking at 38.6 mV K^−^^1^ at 15 wt.% PSS before declining. At 90% RH, the peak *S*_i_ further increased to 44.8 mV K^−^^1^, corresponding to 22% enhancement over pristine PAETC hydrogel (Figure 4b). Meanwhile, *σ*_i_ increased linearly with PSS content, indicating improved ion transport. For comparison, polyethyleneimine (PEI), a branched polycation commonly referred to as a “proton sponge”, was incorporated into the PAETC matrix. The –NH_2_ groups in PEI are readily protonated in neutral aqueous environments due to the high basicity of its tertiary (pKa = 11.6) and secondary (pKa = 6.7) amines [36, 37, 38]. This strong protonation leads to sequestration of free protons, thereby raising the activation energy barrier for proton transport and significantly suppressing *S*_i_ in the PAETC/PEI hydrogel to 8.5 mV K^−^^1^ (Figure S17).

The role of PSS in enhancing proton transport is systematically elucidated across multiple length scales. In PAS hydrogels, PSS chains predominantly adopt the deprotonated form (–SO_3_^−^), enabling ionic complexation with the −(CH_3_)_3_N^+^ groups of PAETC [39, 40], as confirmed by ATR‐FTIR and X‐ray photoelectron spectroscopy (XPS) analyses. The ATR‐FTIR spectra display characteristic bands at 1006 and 1034 cm^−^^1^, corresponding to the symmetric SO_2_ stretching of sulfonic acid groups [41], along with a slight blue shift of the broad high‐frequency peak, indicative of weakened −(CH_3_)_3_N^+^‐water interactions (Figure S18). Incorporation of PSS also causes distinct shifts in the XPS spectra: the O 1s peak moves to higher binding energy, while the N 1s peak shifts to lower energy (Figure S19). To further validate the interaction between −(CH_3_)_3_N^+^ groups and water molecules, ^1^H NMR spectroscopy was performed (Figure S20). In the anhydrous system, the singlet assigned to the −(CH_3_)_3_N^+^ protons appears at 3.15 ppm. Upon the addition of 20 wt.% water, this signal shifts downfield to 3.22 ppm. This deshielding indicates a significant change in the local electronic environment of the quaternary ammonium group, consistent with strong hydration interactions between water molecules and the cationic headgroup. These results support the formation of a hydration shell around −(CH_3_)_3_N^+^, which is expected to facilitate proton release and transport in the hydrated system.

These spectral features collectively reveal acid‐base pairing between –SO_3_^−^ and −(CH_3_)_3_N^+^, which promotes proton diffusion through the formation of ion nanochannels [42, 43]. To further substantiate this mechanism, the diffusion coefficient (*D*) of H_3_O^+^ ions was extracted from mean square displacement (MSD) profiles obtained from MD simulations (Figure S21) [44, 45]. As shown in Figure 4c, the PAS hydrogel containing 10 wt.% PSS (PAS‐10) exhibits a *D* value 3.53 times higher than that of PAS‐5. However, at PSS concentrations above 10 wt.%, *D* decreases progressively, with pure PSS retaining only ∼25% of the maximum value. This sealed‐state trend is consistent with HGP expectations when ∇*a*_w_ is minimized. The decline in *D* at high PSS loading arises from two synergistic effects: 1) excessive –SO_3_^−^ disrupts ordered ion nanochannels, increasing proton‐anion friction and creating energy barriers for vehicle diffusion (inset of Figure 4c), and 2) intensified ion hydration at higher proton concentrations reduces water mobility, limiting Grotthuss‐type proton hopping via the formation of highly polarized water clusters [46, 47, 48, 49].

Another important consequence of PSS incorporation in PAS hydrogels is its influence on the hydration structure, as reflected in their mechanical behavior (Figure S22). The mechanical strength of PAS hydrogels decreases markedly relative to PAETC hydrogels with comparable water content, with PAS‐20 failing to maintain structural uniformity. The tensile stress declines sharply from 595.5 to 11.0 kPa as the PSS fraction increases from 0 to 20 wt.%. Rheological and tensile analyses further reveal a transition from a tough, elastic network to a weaker, more fluid‐like state with rising PSS concentration. In particular, tanδ values for PAS‐15 and PAS‐20 exceed 1.0 at low frequencies, signifying a shift toward viscous‐dominated behavior. This pronounced softening is attributed to reduced heterogeneity within the polymer network, resulting from the extension of coiled polymer chains upon deprotonation of negatively charged –SO_3_^−^ groups (Figure 4d). SAXS further confirms this structural homogenization, showing that the broad characteristic peak at *q* = 4.1 nm^−^^1^ gradually diminishes with increasing PSS content (Figure 4e). Within the HGP framework, this mechanical softening reflects a micro‐to‐mesoscale reconfiguration of the hydration topology, which helps reduce tortuosity and promotes more efficient proton transport paths.

Overall, this structural transition promotes proton transport through two synergistic mechanisms. First, the formation of a continuous, low‐tortuosity channel network eliminates phase‐separated nanodomains (e.g., PNCs structure), thereby reducing interfacial resistance and facilitating long‐range proton migration [50, 51]. As a result, the ion migration energy barrier (*E*_a_) of the PAS‐15 hydrogel is markedly reduced to 7.28 kJ mol^−^^1^, compared with 30.76 kJ mol^−^^1^ for the PAETC hydrogel (Figure 4f). Second, the resulting homogeneous network supports a steeper water concentration gradient under thermal bias, driving directional proton flux from the hot to the cold end. This effect is evidenced by the enhanced evaporation rates observed in PAS hydrogels, particularly PAS‐15, relative to PAETC hydrogels at elevated temperatures (Figure 4g). The increased evaporation reflects optimized water distribution and altered water states within the homogeneous matrix. Notably, intermediate water (IW) exhibits a lower evaporation enthalpy than either free water (FW) or bound water, owing to its weaker intermolecular interactions [52]. Raman spectral analysis (Figure 4h) identifies these water states via band deconvolution into four characteristic peaks: IW (3514/3630 cm^−^^1^) and FW (3233/3401 cm^−^^1^) [53]. The IW/FW ratio in PAS‐15 reaches 0.858, substantially higher than the 0.587 observed in PAETC (Figure S23). This elevated IW fraction signifies an HGP‐compatible matrix capable of sustaining a stronger ∇*a*_w_ ​under open boundaries, thereby enhancing hydration‐driven proton transport.

Taken together, these results identify PSS as a microscopic hydration‐engineering lever within the HGP framework. It increases the local proton concentration, homogenizes and broadens aqueous transport channels, and synergizes with boundary‐programmed ∇*a*_w_ to amplify both the dissociation‐driven (*V*_d_​) and hydration‐enhanced diffusion (*V*_t2_) components. Collectively, these effects establish a hierarchical structure‐function relationship spanning multiple length scales, microscopic proton channels, mesoscopic percolating networks, and macroscopic water gradients, which together create a positive feedback loop for proton transport. Through coordinated programming of composition and boundary conditions, HGP effectively lowers proton‐hopping energy barriers and optimizes directional transport across the material (Figure 4i), enabling PAS hydrogels to achieve record‐high *S*_i_ values among polyelectrolyte‐based iTE systems (Figure S24).

### 2.4 iTE Hydrogel Modules for Low‐Grade Heat Harvesting

**FIGURE 5:**
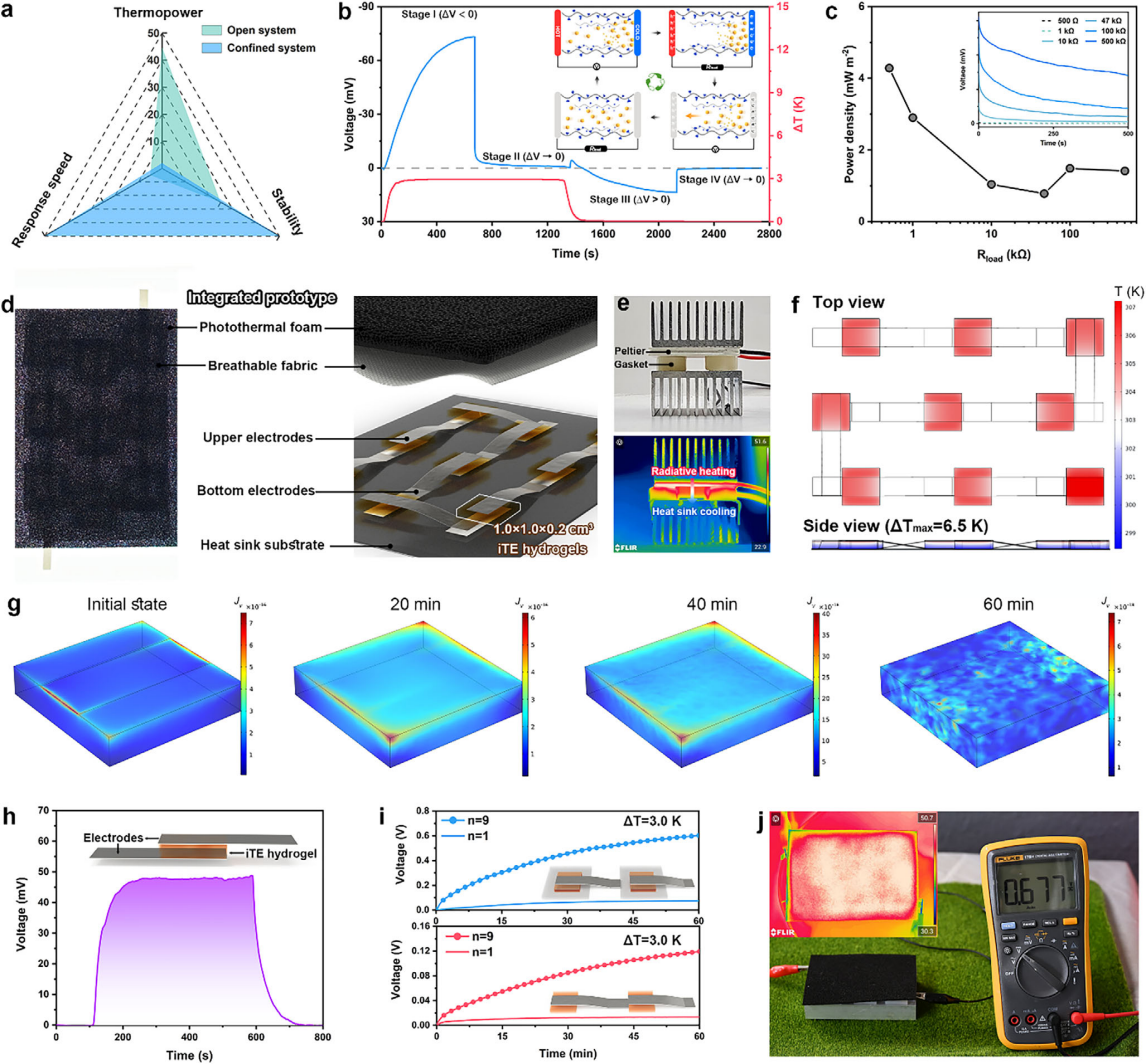
(a) Schematic comparison of iTE hydrogel performance under open and sealed operational environments. (b) Voltage output and Δ*T* profiles under a 10 kΩ external load. (c) Average power density of the ITEC as a function of external load resistance. Inset: voltage decay profiles during stage II. (d) Integrated energy‐harvesting prototype and exploded schematic of its multilayer architecture. (e) Photograph of the testing setup and corresponding IR thermal images. (f) Simulated temperature field of the hydrogel under radiative heating from a 40(C source. (g) Simulated *J*_v_ field of the laterally confined hydrogel under the same heating condition. (h) Time evolution of the open‐circuit voltage under Δ*T* = 3.0 K for series‐connected arrays (*n* = 9) with a small exposed surface area. (i) Open‐circuit voltage evolution under Δ*T* = 3.0 K for single‐element and series‐connected arrays (*n* = 9) with larger exposed areas. Top: laterally confined configuration (top surface open to the ambient). Bottom: unconfined configuration (sidewalls and top surface open to ambient). (j) Photograph of the outdoor solar‐thermal harvester prototype. Inset: IR thermography.

The hydration‐engineering strategy enables ultrahigh thermopower for iTE hydrogels under asymmetric hydration (open) conditions, which is favorable for low‐grade heat harvesting (Figure 5a). The energy harvesting performance of PAS‐15 hydrogels in an open environment was evaluated as an ionic thermoelectric capacitor (ITEC) operating in four stages (Figure 5b; Figure S25). Noticeably, the voltage decay during stage II decreases with increasing external load (*R*_load_), reflecting an extended decay time constant. The average power density (*P*_a_) delivered to the external load was calculated as follows [54],

(4) $$P_{a}=\frac{1}{A\Delta t}\int \frac{V^{2}dt}{R_{\mathrm{load}}}$$

where *V* is the output voltage across the external load during a thermal cycle, *A* is the cross‐sectional area of the iTE material, and Δ*t* is the combined duration of stages II and IV. A maximum average power density of 4.28 mW m^−^^2^ was achieved with a 500 Ω load, which is comparable to other reported high‐performance ITECs (Figure 5c) [55, 56].

To translate the material‐level advantages into a practical energy‐harvesting device, we constructed an integrated stack comprising a photothermal foam layer, a breathable fabric, top and bottom electrodes, iTE hydrogel elements, silicone molds, and a heat‐sink substrate (Figure 5d; Figure S26). The photothermal foam efficiently absorbs broadband light and converts it into heat, while the fabric layer allows vapor transport, preserving the asymmetric hydration state at the hydrogel surface. The hydrogel elements (∼1.0 × 1.0 × 0.2 cm^3^) are arranged in a 3 × 3 array and connected via printed interconnects. This multilayer architecture enhances light‐to‐heat conversion near the exposed surface, mitigates lateral heat loss through the electrode‐spacing design, and stabilizes the through‐thickness thermal gradient by coupling to the heat‐sink substrate. Photographs and IR thermography confirm a robust vertical thermal gradient under a non‐contact radiative heating setup (Figure 5e). In this configuration, the heat source is spatially separated from the top surface, while the backside is coupled to a finned heat spreader that acts as a cold reservoir, maintaining a consistent hot‐cold separation across the stack without impeding evaporation. Simulated thermal maps under radiative heating from a 40°C source reveal a uniform temperature distribution across the surface and a concentrated through‐thickness gradient within the hydrogel/heat‐sink region (Figure 5f). The maximum temperature difference (Δ*T*_max_) reaches 6.5 K, sufficient to drive thermovoltage generation within the iTE hydrogel.

The device geometry and architecture were optimized not only to establish a vertical temperature gradient but also to maintain a stable water gradient essential for hydration‐controlled thermovoltage generation (Figure 5g). Coupled evaporation–diffusion–heat simulations and voltage measurements reveal that when the top electrode covers most of the hydrogel surface, vapor exchange is severely restricted, causing the total water vapor mass flux (*J*_v​_) to rapidly decay to near zero (Figure S27). Consequently, the thermovoltage of a nine‐element series array quickly saturates at 0.048 V under Δ*T* = 3.0 K (Figure 5h). Expanding the exposed top area restores vapor exchange, allowing ∇*a*_w_​ to develop gradually. In this configuration, the simulated *J*_v_ field evolves from edge‐biased to uniformly top‐down over 60 min, consistent with a prolonged voltage rise and an increased output of 0.12 V (*n* = 9) (Figure 5i, bottom). Further improvement is achieved by adopting a laterally confined design with sealed sidewalls, which forces vapor to escape solely from the top surface, maximizes hydration asymmetry, and yields a high thermovoltage of ∼0.6 V (*n* = 9) (Figure 5i, top). The corresponding single‐element maximum current and power densities reach 0.3 A m^−^^2^ and 8.35 mW m^−^^2^, respectively (Figure S28). Finally, coupling the laterally confined element with a photothermal foam sustains a persistent vertical hydration–temperature bias under outdoor illumination. IR thermography confirms localized surface heating, while the breathable top layer maintains a stable water‐activity profile that cooperates with Δ*T*, generating a thermovoltage of 0.677 V (Figure 5j; Supplementary Movie S1). These results demonstrate ambient‐light‐to‐thermovoltage conversion through hydration‐engineered pathways in the integrated prototype.

### 2.5 Thermal‐Tactile Perception for a Robotic Hand

**FIGURE 6:**
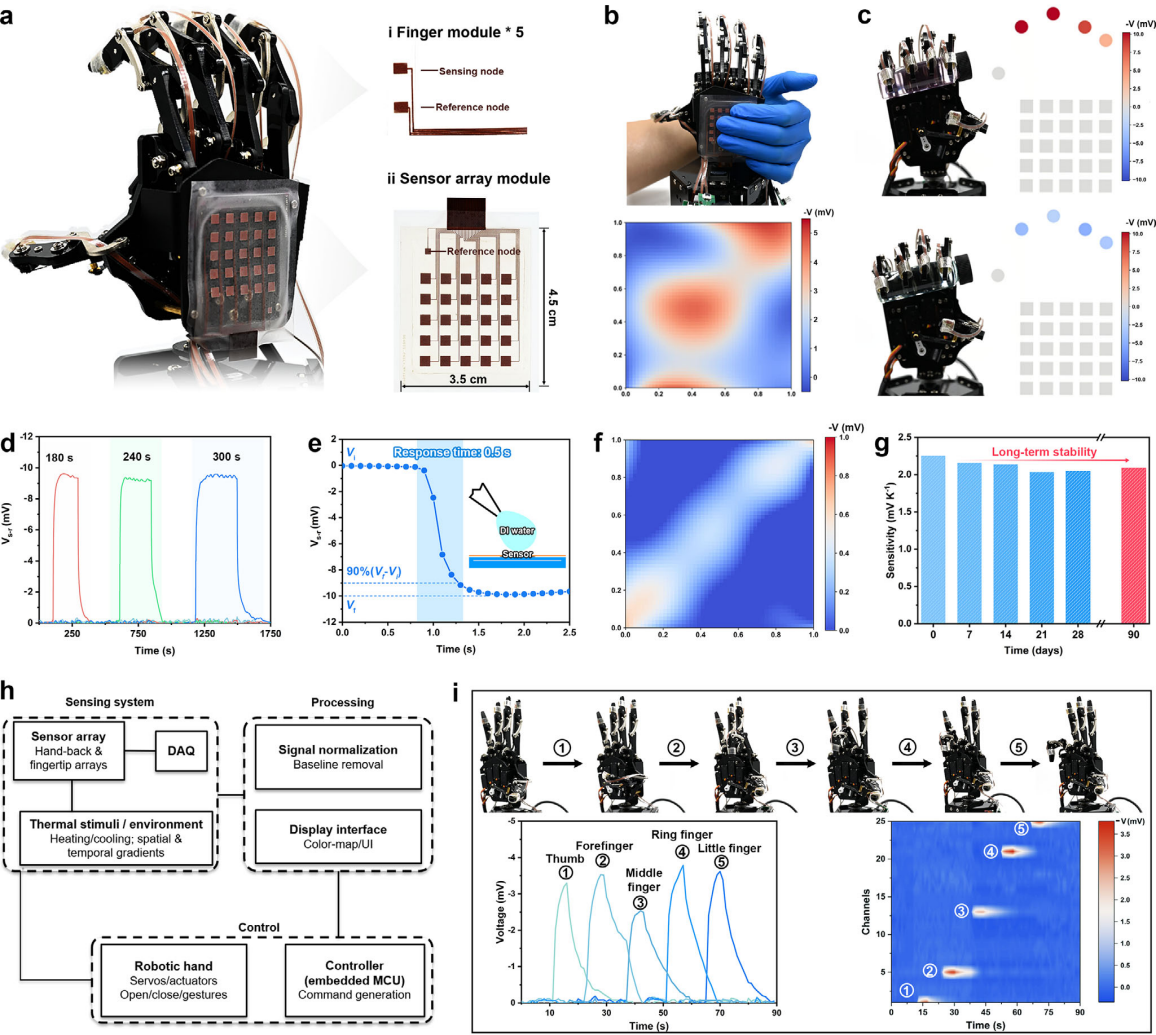
(a) Photograph of a robotic hand integrating five finger modules and a hand‐back thermal sensor array. Right: schematic layouts of an individual finger module and the 2D sensor array. (b) Representative thermogram of the hand‐back array during human‐robot contact. (c) Responses of the five finger modules when grasping hot‐ (top) and cold‐water bottles (bottom), accompanied by corresponding thermograms. (d) Voltage traces from selected sensing nodes under prolonged heating for durations of 180, 240, and 300 s. (e) Dynamic response of a sensing node to a transient thermal pulse. (f) Thermal‐trace voltage colormap recorded by the 2D array as a moving heat source scans across the surface. (g) Long‐term sensitivity stability over 90 days. Devices were stored sealed and at rest between measurements (20‐25°C, 60–80% RH) and tested at scheduled time points. (h) System architecture of the smart thermal‐sensing robotic hand. (i) Thermally triggered gesture sequences. Top: key video frames; bottom: corresponding voltage profiles and map.

The rapid proton diffusion kinetics and stable hydration structure of the iTE hydrogel under sealed conditions provide distinct advantages for thermal sensing applications. To demonstrate this capability, we integrated five finger modules and a hand‐back sensor array, with the PAETC hydrogel serving as the active sensing element, into a robotic hand system (Figure 6a). Each finger module incorporates a sensing node referenced to a local ground, while the hand‐back array functions as a 2D pixelated thermal sensor with a footprint of approximately 3.5 × 4.5 cm^2^. The modules are connected to a lightweight data‐acquisition and control unit enabling real‐time temperature mapping. Spatial thermography confirms that the hand‐back array accurately captures temperature distributions generated by human‐robot contact (Figure 6b). Simultaneously, each fingertip sensor reliably reports the temperature of grasped objects, distinguishing hot and cold stimuli during single‐touch interactions (Figure 6c). Localized heating and cooling appear as red and blue lobes in the voltage maps, providing an intuitive, spatially resolved visualization of thermal patterns. Importantly, to confirm that these voltage patterns are dominated by thermal rather than pressure stimuli, we conducted an isothermal pressure‐control test (Δ*T* ≈ 0), which showed negligible voltage changes under stepwise loading (Figure S29).

The fabricated thermal sensors exhibit outstanding performance characteristics, including high signal stability, rapid response, and long‐term operational stability. Under steady thermal stimulation, the sensor maintains an exceptionally stable voltage plateau for over 300 s (Figure 6d). Kinetic measurements further reveal fast protonic heat‐to‐charge transduction, with a 90% response time of ∼0.5 s in response to dynamic thermal pulses (Figure 6e). This fast response enables real‐time tracking of moving heat sources. The array reconstructs their spatiotemporal trajectories as continuous ridges in the channel‐time map (Figure 6f; Figure S30 and Supplementary Movie S2), which can be directly used to trigger programmable control sequences. Long‐term testing under sealed conditions confirms stable sensitivity over extended periods (weeks to months), demonstrating that the confined hydration environment effectively suppresses evaporation‐induced signal drift (Figure 6g; Figure S31). Collectively, these results underscore the potential of the flexible iTE‐hydrogel‐based thermal sensor array for advanced, real‐time thermal mapping and intelligent sensing applications (Figure S32 and Table S1).

To close the perception‐action loop, we implemented the sensing‐to‐control pipeline illustrated in Figure 6h, integrating sensing, signal processing, and control modules. This system translates thermal inputs directly into gesture‐level robotic behaviors. As demonstrated in Figure 6i and Supplementary Movie S3, sequential thermal interactions trigger distinct robotic gestures, showcasing effective conversion of thermal cues into coordinated motion. The temporal signal traces and channel‐resolved colormaps are co‐registered, linking stimulus, perception, and actuation in real time. Together, these results establish sealed‐mode protonic hydrogels as fast, stable, and spatially resolved thermal sensors, and highlight their seamless interoperability with robotic manipulators for thermal‐tactile human‐machine interaction.

## 3 Conclusion

This work establishes HGP as a general design principle for ionic thermoelectrics, in which programmable hydration pathways reconcile the conventional trade‐off among thermopower, response speed, and operational stability. By tailoring hydration across micro‐, meso‐, and macro‐scales, PAETC‐based hydrogels can function in two complementary regimes. In open systems, heterogeneous hydration induces asymmetric proton dissociation coupled with a sustained water‐activity gradient, yielding high thermopower in pristine PAETC. In contrast, sealed systems suppress evaporation, resulting in symmetric hydration that facilitates rapid proton redistribution and subsecond response, albeit with a reduced voltage amplitude. Further compositional engineering with PSS homogenizes the hydration landscape and activates multiscale proton‐transport synergy, enhancing *S*_i_ to 44.8 mV K^−^^1^ under open operation. Within this framework, water serves dual and interconnected roles as a proton donor through solvation shells and as a structural architect shaping hydration‐modulated transport pathways, together enabling intrinsic reconfigurability. Translating these mechanisms into functional devices, we demonstrate a hydro‐thermal dual‐gradient harvester delivering ∼0.6 V at Δ*T* = 3.0 K, as well as a sealed‐mode thermal‐tactile interface capable of mapping temperature fields, tracking spatiotemporal trajectories, and triggering gesture‐level robotic actuation. Looking ahead, HGP offers a scalable and versatile strategy for designing next‐generation iTE systems, bridging energy harvesting and perceptive control within a unified material platform.

## 4 Experimental Section

### 4.1 Materials

(2‐(Acryloyloxy)ethyl)trimethylammonium chloride solution (AETC, 80 wt.% in water), 2‐hydroxy‐2‐methylpropiophenone (HMPP, 97%), bis(trifluoromethane)sulfonimide lithium salt (Li[TFSI]), polystyrene sulfonic acid (PSS) solution (30 wt.% in water), and polyethyleneimine (PEI) were all purchased from Sigma‐Aldrich and used as received without further purification. All experiments were conducted using deionized (DI) water with a resistivity greater than 18.2 MΩ cm.

### 4.2 Synthesis of PAETC and PAETC/PSS Hydrogels

PAETC hydrogels were prepared via a one‐step radical polymerization. Briefly, 4.84 g of AETC solution (20 mmol) was thoroughly mixed with 1.0 µL of diluted HMPP solution (40 µL mL^−^^1^ HMPP in ethanol) to achieve homogeneity. The precursor mixture was then exposed to UV irradiation (365 nm, 20 W) for 30 min to initiate polymerization and form the hydrogel.

PAETC/PSS hydrogels were prepared by mixing 4.84 g of AETC solution (20 mmol) with varying amounts of PSS solution (0.68, 1.43, 2.28, and 3.22 g) and 1.0 µL of diluted HMPP solution, resulting in homogeneous mixtures with AETC:PSS weight ratios of 95:5, 90:10, 85:15, and 80:20. The mixtures were then subjected to UV polymerization to yield hydrogels, designated as PAS‐5, PAS‐10, PAS‐15, and PAS‐20, respectively.

The water content of the resulting PAETC or PAETC/PSS hydrogels was controlled by equilibrating samples under different humidity conditions.

### 4.3 Synthesis of P[AET][TFSI] Polymers

The [AET][TFSI] monomer was synthesized using an anion‐exchange method. Typically, 2.5 g of AETC solution was mixed with 10 mL of aqueous Li[TFSI] solution (1 mol L^−^^1^) and stirred vigorously at 25°C for 2 hrs. After phase separation, the lower oil layer was collected and washed five times with deionized (DI) water. The transparent [AET][TFSI] liquid was obtained by drying the solution at 70°C for 12 hrs. The preparation of P[AET][TFSI] followed the same procedure as for PAETC, through polymerization under UV light (365 nm, 20 W) for 30 min.

### 4.4 Synthesis of PAETC/PEI Hydrogels

To prepare PAETC/PEI hydrogels, 4.84 g of AETC solution (20 mmol), varying amounts of PEI solution (0.20, 0.43, 0.97, 1.66, and 2.58 g), and 1.0 µL of HMPP were thoroughly mixed to form homogeneous solutions with different AETC:PEI weight ratios (95:5, 90:10, 80:20, 70:30, and 60:40). The PAETC/PEI hydrogels were obtained after polymerization under UV light (365 nm, 20 W) for 30 min.

### 4.5 Materials Characterization

The chemical structure of the samples was analyzed using attenuated total reflectance Fourier transform infrared spectroscopy (ATR‐FTIR, VERTEX 70v), X‐ray photoelectron spectroscopy (XPS, ESCA PHI 5400), Nuclear magnetic resonance spectra (NMR, Bruker Avance III HD 400 MHz), and Raman spectroscopy (Alpha300R). The thermal stability of the ionic liquid was evaluated by thermogravimetric analysis (TGA, HITACHI STA300). In situ ATR‐FTIR measurements were performed to investigate the effect of temperature on the states of water in the PAETC hydrogels. The hydrogel sample was placed in a sealed reaction chamber with dry air introduced from the bottom and vented from the top. The chamber was heated from 24°C to 50°C at a rate of 0.5°C min^−^^1^. Each spectrum was collected with 32 scans at a resolution of 4 cm^−^^1^.

### 4.6 Thermopower Measurement

Ionic thermopower measurement of the hydrogel sample was conducted using a custom‐built apparatus comprising a glass backplane, Peltier plates, Ag electrodes, and thermocouples. The open‐circuit voltage between the two Ag electrodes was recorded under varying temperature gradients. The ionic thermopower was calculated using:

(5) $$S_{i}=-\;\frac{\Delta V}{\Delta T}=-\frac{V_{\mathrm{hot}}-V_{\mathrm{cold}}}{T_{\mathrm{hot}}-T_{\mathrm{cold}}}$$

where Δ*V* is the voltage difference between the hot and cold ends, Δ*T* is the temperature difference measured by T‐type thermocouples between two Ag electrodes (spacing: ∼1.0 mm). Voltage was measured using a Keithley 2400 source meter. Hydrogel thickness was determined with a micrometer.

### 4.7 Ionic Conductivity Measurement

The ionic conductivity of the iTE hydrogels in the relaxed state was determined by the AC impedance spectroscopy using an Autolab potentiostat galvanostat. The hydrogel was sandwiched between two stainless steel plates. The voltage amplitude was 10 mV, and the frequency was scanned from 100 kHz to 1 Hz. The ionic resistance was obtained by extrapolating the curve with the real‐axis intercept. The ionic conductivity was then calculated as

(6) $$\sigma_{i}=\frac{L}{A\;\cdot \;R_{i}}\;$$

where *L* is the separation between the two electrodes, *R*_i_ is the ionic resistance, and *A* is the area.

By measuring the ionic conductivity at different temperatures, the ion migration energy barrier (*E*_a_) was obtained according to the Arrhenius equation:

(7) $$\sigma_{i}=Aexp\left(-\frac{E_{a}}{RT}\right)$$

where *A* is the pre‐constant, *T* is the absolute temperature, and *R* is the gas constant.

## Supporting information




**Supporting File 1**: advs75158‐sup‐0001‐SuppMat.docx.

**Supporting File 2**: advs75158‐sup‐0002‐MovieS1.mp4.

**Supporting File 3**: advs75158‐sup‐0003‐MovieS2.mp4.

**Supporting File 4**: advs75158‐sup‐0004‐MovieS3.mp4.
