# Supplementary material for: Programmable Hydration Pathways Enable Reconfigurable Ionic Thermoelectrics for Energy Harvesting and Thermal‐Tactile Interaction
Source: Adv Sci (Weinh). 2026 Apr 7;13(36):e75158. doi: 10.1002/advs.75158 (PMC13317698; doi:10.1002/advs.75158)
Supplement: Supplementary file 1 — Supporting File 1: advs75158‐sup‐0001‐SuppMat.docx. [file ADVS-13-e75158-s001.docx]

Supporting Information

**Programmable hydration pathways enable reconfigurable ionic thermoelectrics for energy harvesting and thermal-tactile interaction**

*Zehao Zhao^#^, Yun Shen^#^, Dongyan Xu**

Department of Mechanical and Automation Engineering, The Chinese University of Hong Kong, Shatin, New Territories, Hong Kong Special Administrative Region, China.

* Corresponding author. Email: dyxu@mae.cuhk.edu.hk (D.X.)

^#^ Contributed equally to this work.

**Experimental**

**Fabrication of the integrated energy harvesting prototype**

The integrated energy harvesting prototype was fabricated by first screen-printing silver conductive ink on a PET substrate to form bottom electrodes and interconnects, followed by in-situ polymerization of the iTE hydrogel within a 1 × 1 × 0.2 cm^3^ mold positioned over each electrode. After curing, a top electrode was gently laminated onto the hydrogel surface to ensure intimate contact. The hydrogel was then overlaid with a breathable fabric and capped with carbon foam to provide photothermal absorption while maintaining vapor permeability. For backside thermal management, a graphite heat-spreader tape was adhered to the underside of the PET substrate. When required, the perimeter of the hydrogel element was sealed to control evaporation, and multiple units were arrayed and interconnected in series to achieve the desired output.

**Fabrication of the iTE hydrogel thermal sensors**

Flexible iTE hydrogel thermal sensors were produced in two formats: finger module with a flexible printed circuit board (FPCB) carrying one sensing node and one reference node, and 2D array module with an FPCB carrying 26 nodes (25 sensing nodes and 1 reference node). The iTE precursor solution was drop-cast into the recessed cavities of the spacer and photo-polymerized under UV light. The completed assemblies were then hermetically encapsulated by TPU lamination to provide mechanical protection and environmental isolation.

**Tensile tests**

Tensile tests were performed at room temperature (25 °C, 60% RH) using a universal testing machine (Instron 4411). Free-standing dumbbell-shaped specimens, with a thickness of approximately 4.0 mm, were prepared using a standard PTFE mold. The films were mounted using pneumatic grips, and the gauge length was set to 1.0 cm. Uniaxial tension was applied at a strain rate of 100 mm min⁻^1^ until rupture. Cyclic stress-strain measurements of PAETC hydrogels were conducted for six consecutive cycles at loading and unloading rates of 300 mm min⁻^1^, with no pauses between cycles.

**Rheological behavior tests**

Dynamic rheological measurements were performed using an MCR 302 rheometer (DHR-3, TA Instruments) equipped with a 20 mm diameter parallel plate. All tests were conducted at 25 °C, and a solvent trap was used throughout to minimize solvent evaporation. Frequency sweep tests were carried out at a fixed strain of 1% over a frequency range of 0.1 to 100 Hz.

**Water content measurement**

The water content of the hydrogels was determined by comparing their weights before and after drying. Samples were heated at incremental temperatures between 24°C and 100°C, with each temperature step maintained for 60 min. The samples were weighed immediately after removal from the heat source. The initial weight (*m*_1_) and the weight after drying at each temperature step (*m*_2_) were recorded using an analytical balance. Water content was calculated as [(*m*_1_ − *m*_2_)/*m*_1_] × 100%. The final value represents the average of five independent measurements.

**Small-angle X-ray scattering (SAXS)**

SAXS measurements were performed using a Xeuss 3.0 SAXS/WAXS system with an X-ray wavelength of 1.54189 Å. Data were collected with an Eiger2R 1M detector (pixel size: 75 μm). SAXS data analysis was carried out using Fit 2D software (European Synchrotron Radiation Facility). Two-dimensional (2D) SAXS patterns were azimuthally integrated to produce one-dimensional (1D) scattering profiles as a function of the scattering vector.

**Molecular dynamics (MD) simulations**

MD simulations were conducted using the Forcite module in Materials Studio 2018 to predict the radial distribution function (RDF) and ion diffusion properties of the hydrogels. Systems consisting of PAETC, PSS, water molecules, and hydronium ions were constructed in a cubic simulation box, with the water mass fraction set to 30 wt% for each sample. Periodic boundary conditions were applied in all three dimensions, and the cutoff distance for long-range interactions was set to 12.5 Å. To remove any persistent metastable states, 25 cycles of quench-annealing dynamics were performed by slowly cycling the system temperature between 298 K and 894 K. Each system was subsequently equilibrated in the NPT ensemble at 298 K and 1 atm for 500 ps, followed by a 2 ns production run in the NVT ensemble. Full simulation trajectories were used to calculate RDFs and mean square displacement (MSD) curves.

**Finite element analysis (FEA)**

COMSOL Multiphysics was employed to simulate the temperature distribution of the device under radiative heating from a 40°C source. A three-dimensional (3D) model was constructed based on the actual device geometry, comprising nine hydrogel units (1 × 1 × 0.2 cm^3^) connected in series and arranged according to the experimental layout. The ambient environment was maintained at 293.15 K (20°C), while the top surface of the device was exposed to radiation from a 40°C source to emulate external thermal excitation.

For the hydrogel evaporation simulation, we established a 3D air‐chamber model containing a hydrogel pad (1 × 1 × 0.2 cm^3^) centrally positioned on the bottom plate, with all dimensions matching the actual element size. The model simultaneously solved transient heat transfer within the solids (hydrogel, electrode, and mold) and moisture transport in the surrounding air, assuming a prescribed laminar airflow with defined inlet and outlet pressures. A radiative environment at 40°C was applied, together with convective heat and mass exchange at the air-gel interface. Evaporation at the gel surface was represented as a mass-flux boundary condition, driven by the difference between surface saturation and ambient vapor concentrations, and thermally coupled to the system as a latent-heat sink. The inlet air was set to a temperature of 313.15 K, relative humidity of 75%, and velocity of 0.1 m s^−1^), while the solid components were initialized at an ambient temperature of 273.15 K. The total mass loss was calculated via surface-integrated flux over the gel boundaries.

**Figure**


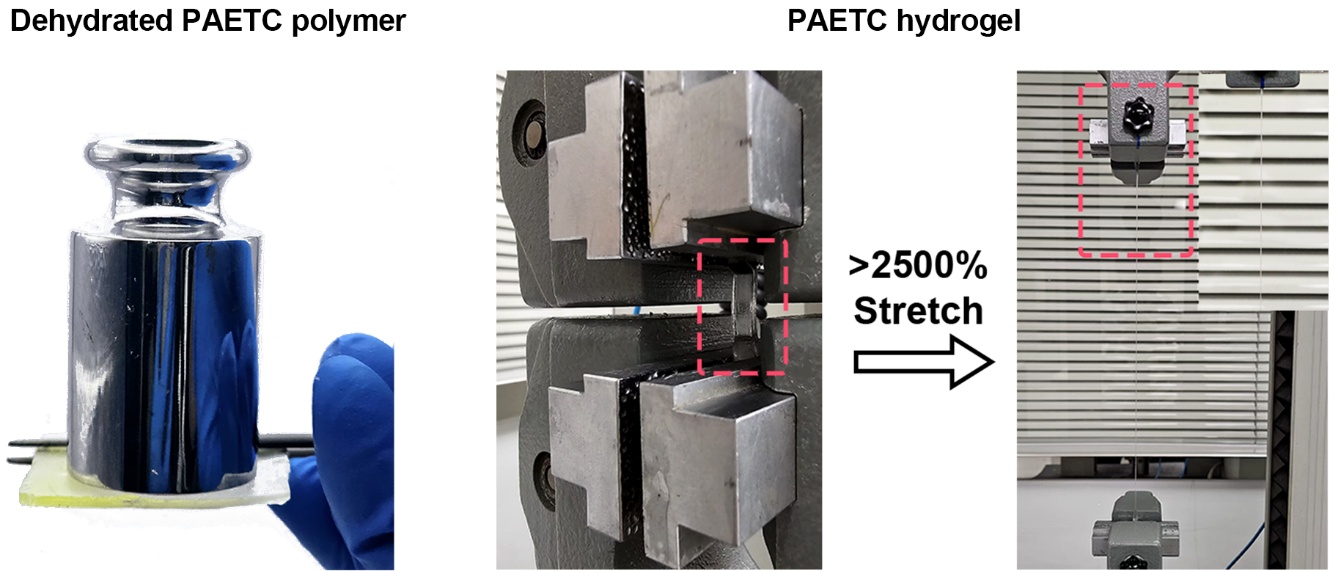


Figure S1. Photographs showing mechanical response of PAETC polymer and hydrogel.

Figure S2. Tensile stress-strain curve of PAETC hydrogel at 15% RH.


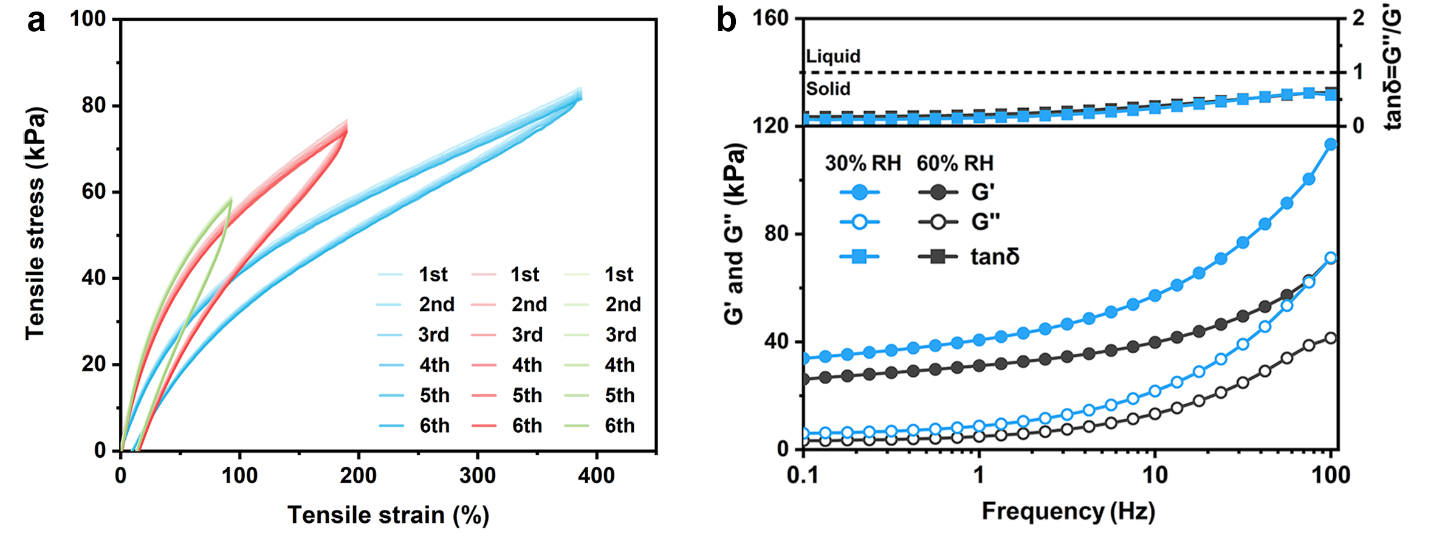


Figure S3. (a) Cyclic stress-strain curves of PAETC hydrogels subjected to strains of 100%, 200%, and 400% over six cycles. (b) Rheological properties of PAETC hydrogels, showing storage (*G*′) and loss (*G*″) moduli, and tan δ.


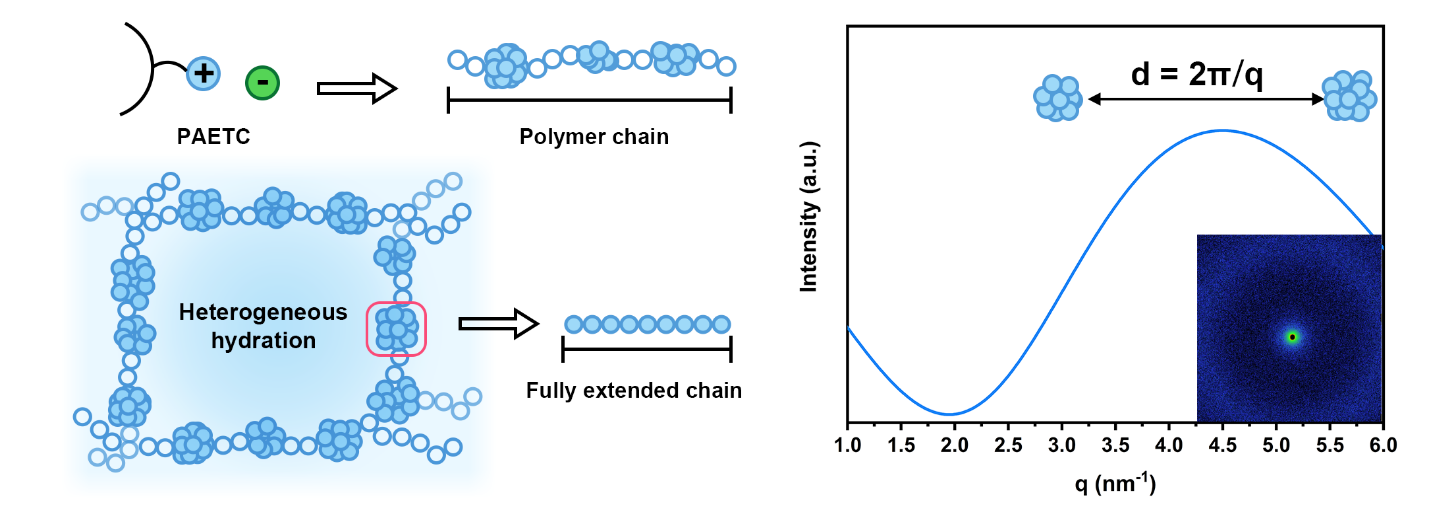


Figure S4. Schematic illustration of the PNCs structure in PAETC hydrogel.


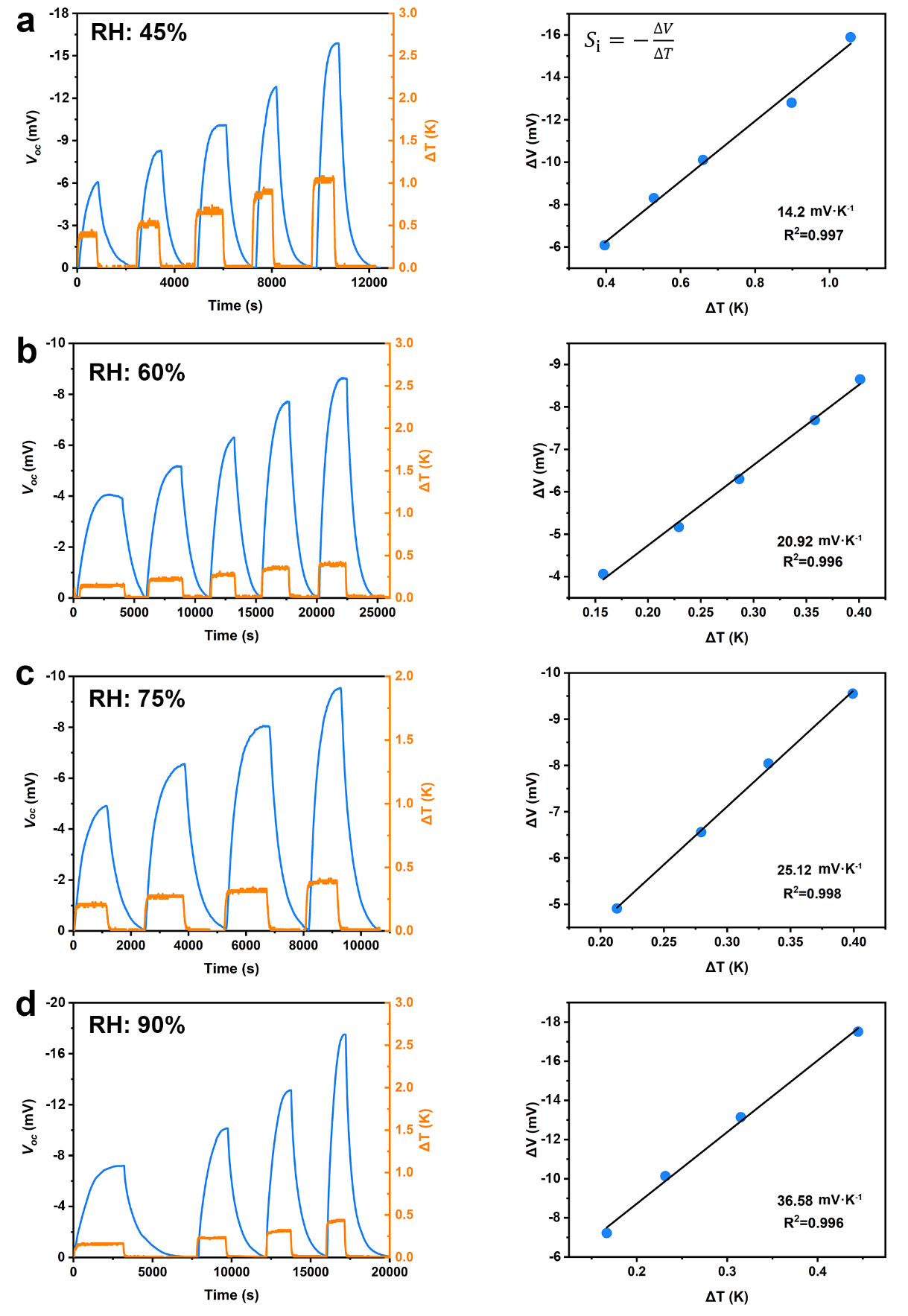


Figure S5. Open-circuit voltage (*V*_oc_) and temperature difference (Δ*T*) profiles of PAETC hydrogels at different RH levels: (a) 45%, (b) 60%, (c) 75%, and (d) 90%. Thermopower is extracted through linear fitting of the Δ*V*−Δ*T* data.

Figure S6. Ionic conductivity of PAETC polymer and hydrogels at different RH levels.


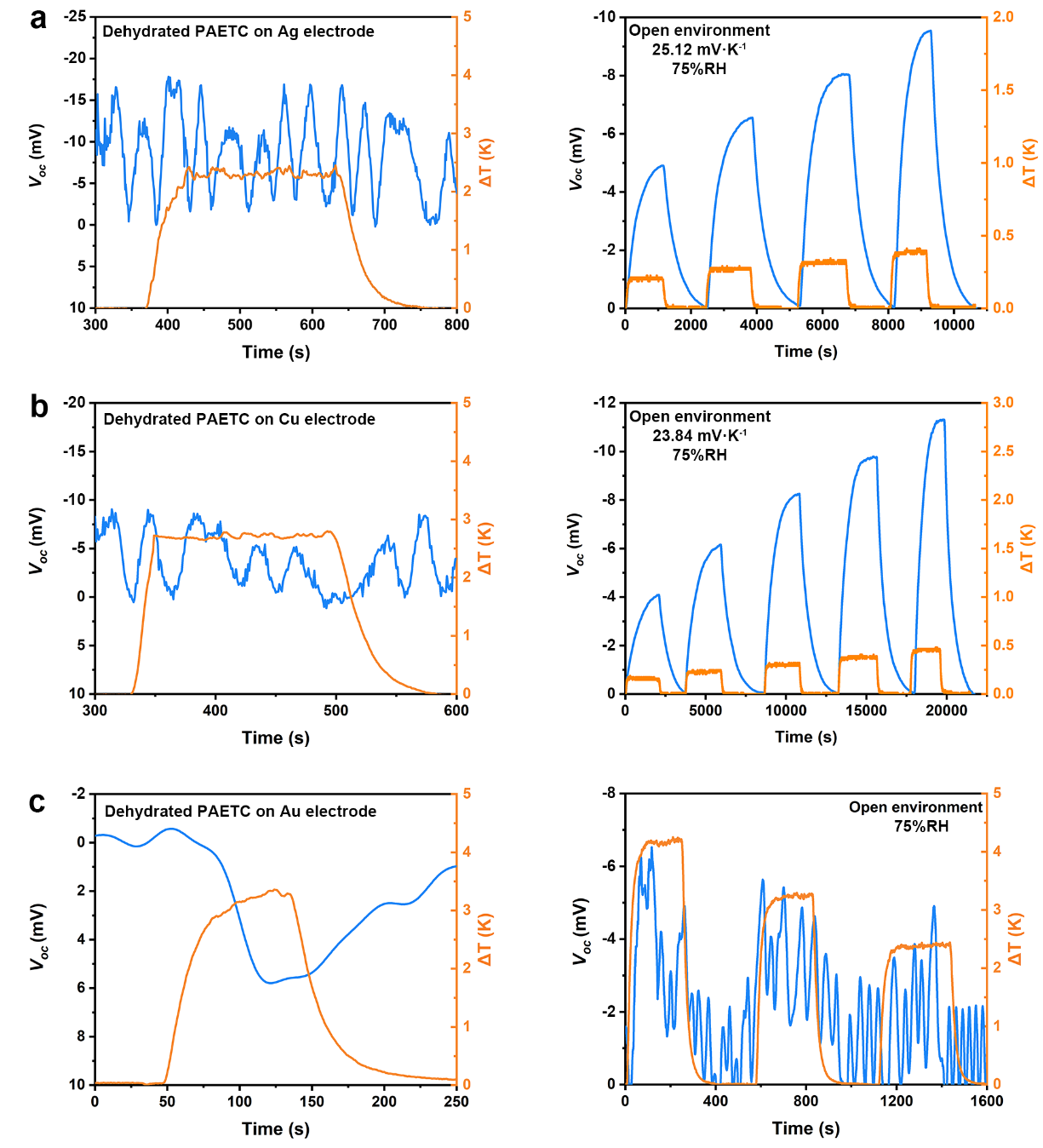


Figure S7. *V*_oc_ and Δ*T* profiles of anhydrous PAETC polymer and PAETC hydrogel at 75% RH on different metal electrodes: (a) Ag, (b) Cu, and (c) Au.


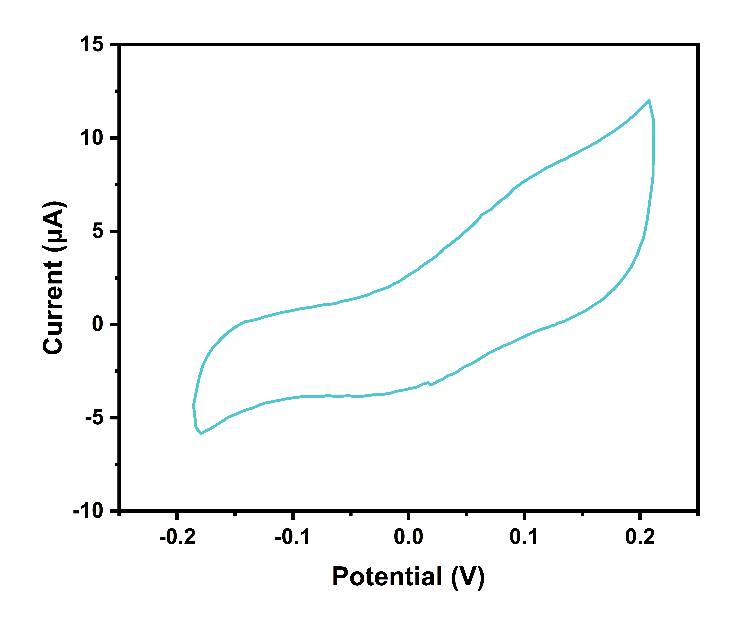


**Figure S8.** Cyclic voltametric current response of a symmetric Ag | gel | Ag two-electrode cell recorded over the investigated voltage window.


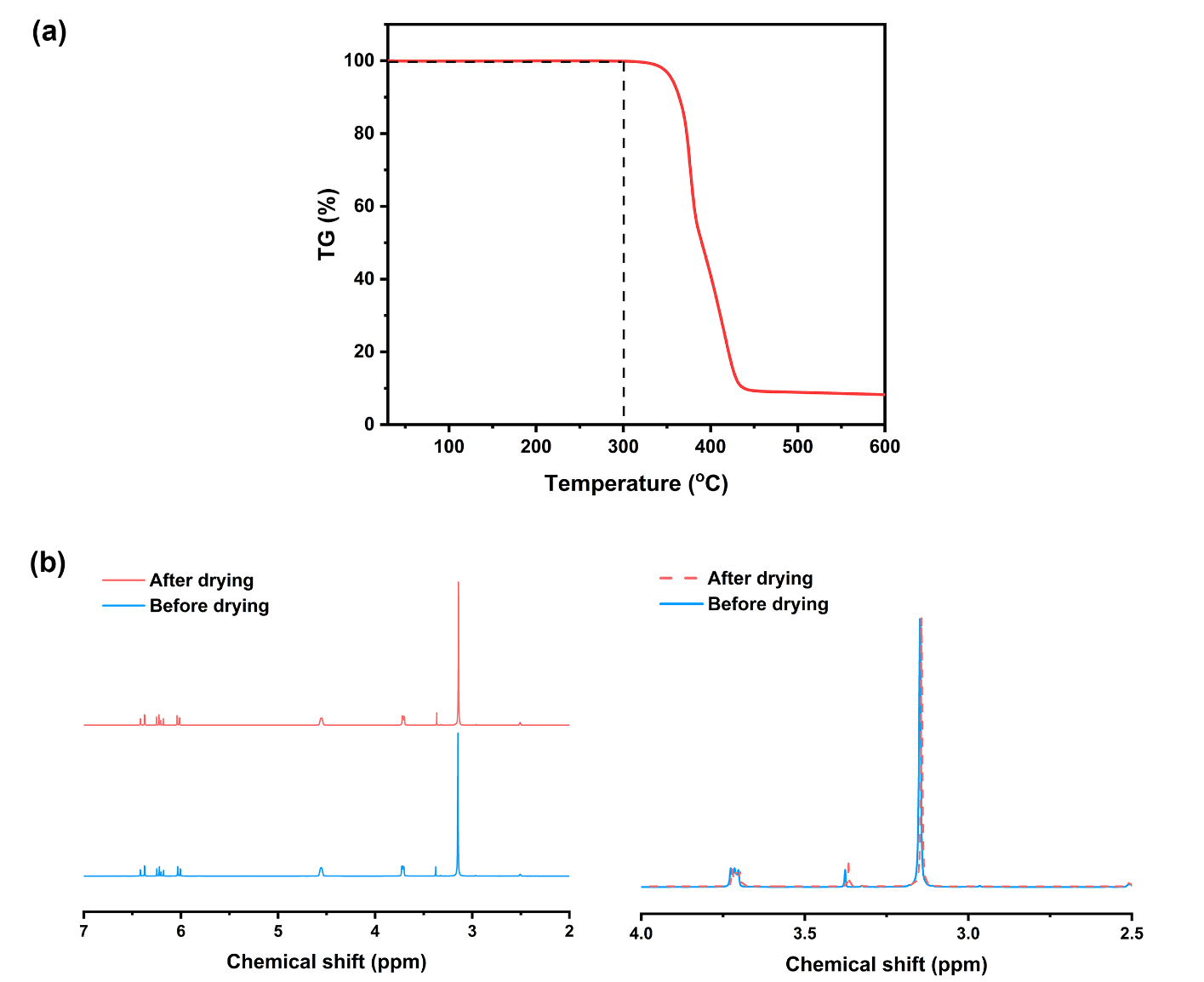


**Figure S9.** (a) TGA curve and (b) ^1^H NMR spectra of [AETC][TFSI] before and after drying.

To ensure the structural integrity of the synthesized ionic liquid under thermal treatment, the thermal stability of [AETC][TFSI] was evaluated by TGA and ^1^H NMR spectroscopy before and after drying (70 °C for 12 hrs). The TGA results show a high decomposition temperature (> 300 °C), while the identical ^1^H NMR spectra before and after drying indicate the absence of thermal degradation, confirming that the chemical framework of [AETC][TFSI] remains stable without detectable structural changes.


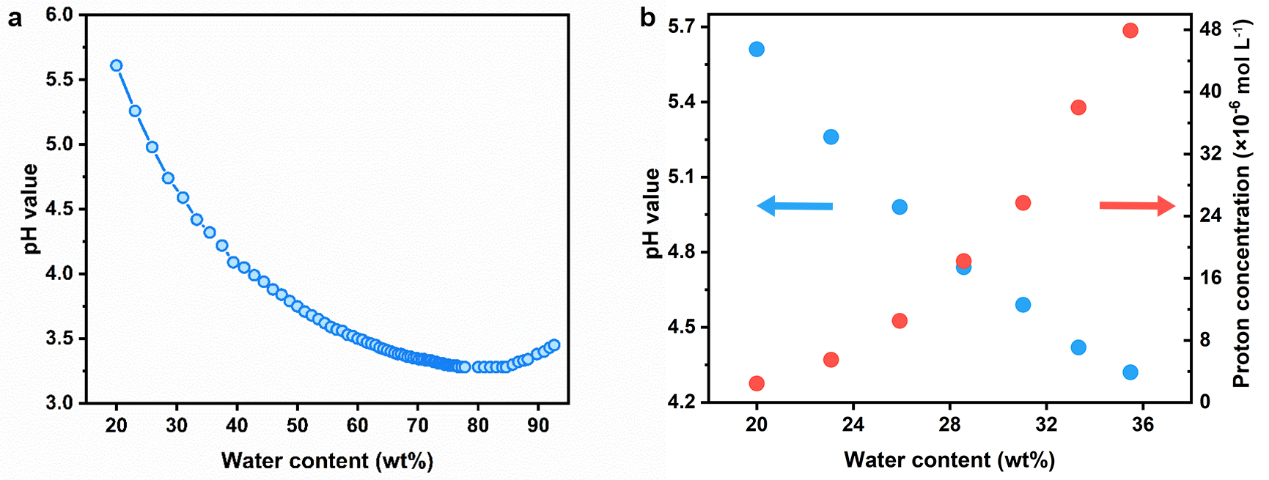


Figure S10. Dependence of the pH value on water content for AETC solutions.


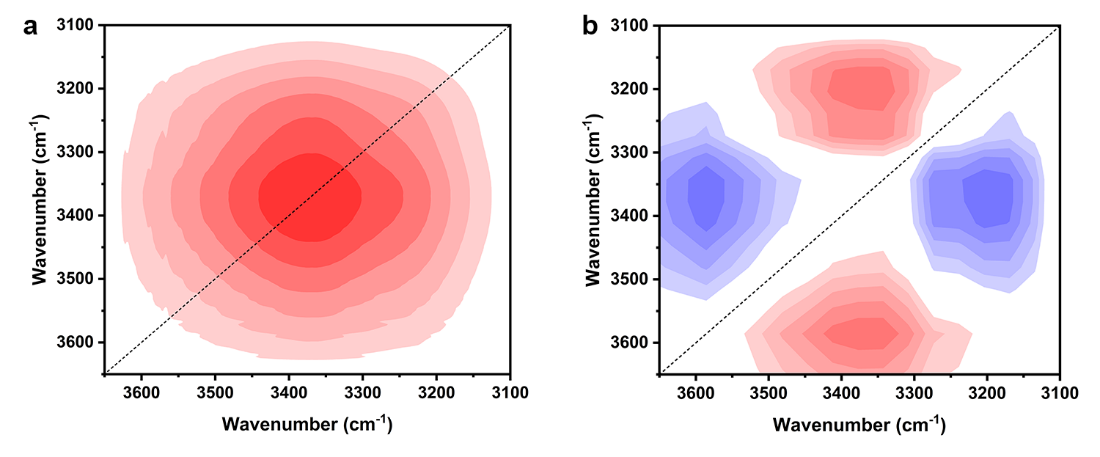


Figure S11. (a) Synchronous and (b) asynchronous contour maps. To monitor the water absorption process in situ, samples were first vacuum-dried for 2 days and then exposed to air at 40% RH, with ATR-FTIR spectra collected every 5 min. Synchronous and asynchronous contour maps were generated using Origin 2021 software. Linear baseline corrections were applied in the 3650-3100 cm⁻^1^ region. In the 2D correlation FTIR spectra, blue and red cross-peaks represent negative and positive correlations, respectively. A strong, positive autopeak in the synchronous spectrum indicates enhancement of the O–H stretching band as water diffuses into the sample. The asynchronous spectrum shows a negative cross-peak at 3565/3364 cm⁻^1^ and a positive cross-peak at 3364/3189 cm⁻^1^, revealing that the water stretching band splits into three distinct components at 3565, 3364, and 3189 cm⁻^1^. According to Noda’s rules, the sequential changes of these bands correspond to 3364 cm⁻^1^ (intermediate water) → 3565 cm⁻^1^ (free water), 3189 cm⁻^1^ (bound water).


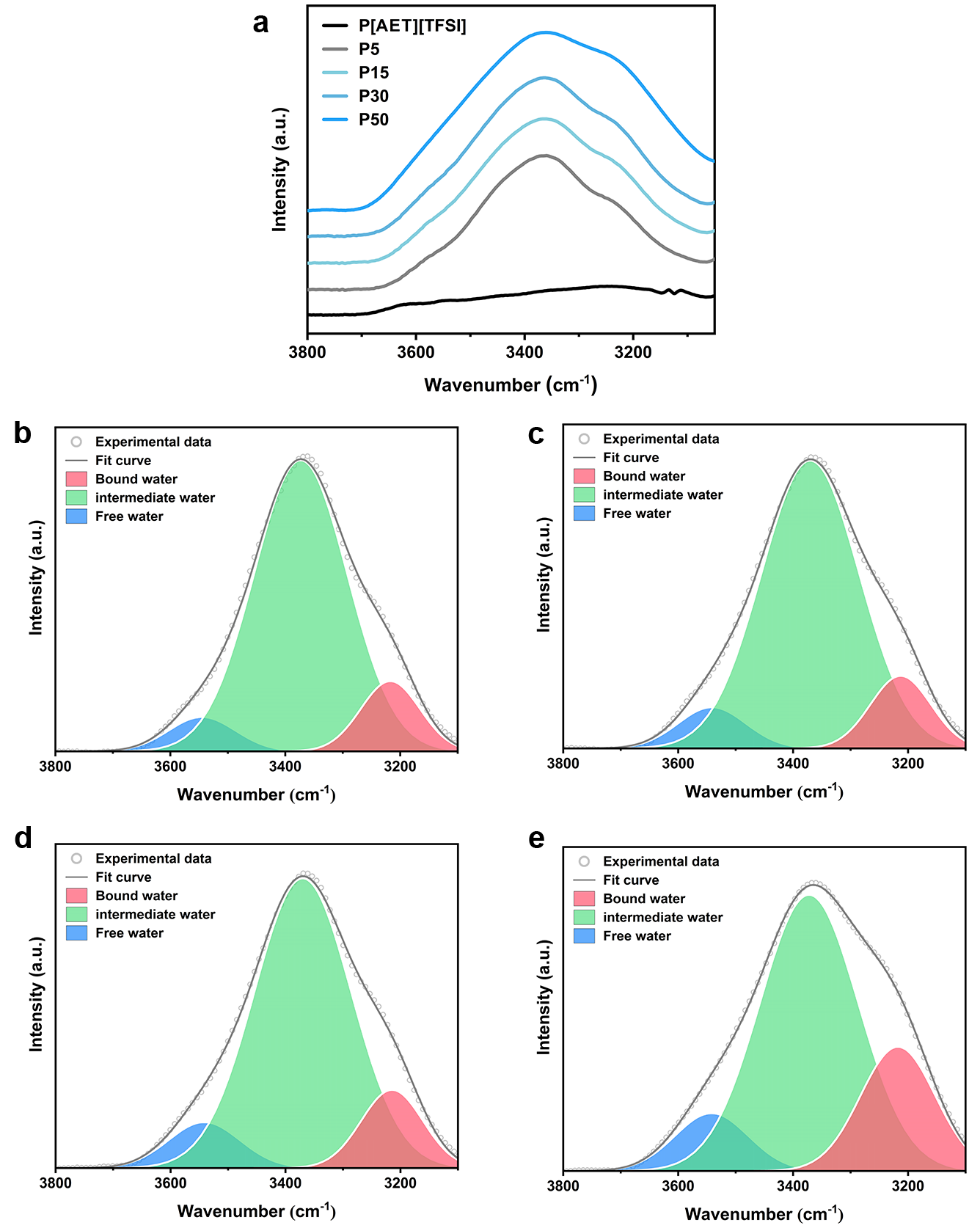


Figure S12. (a) ATR-FTIR spectra of P[AET][TFSI] and PAETC hydrogels with different water contents (P5, P15, P30, and P50). Fitted FTIR spectra in the high-frequency region for (b) P5, (c) P15, (d) P30, and (e) P50.


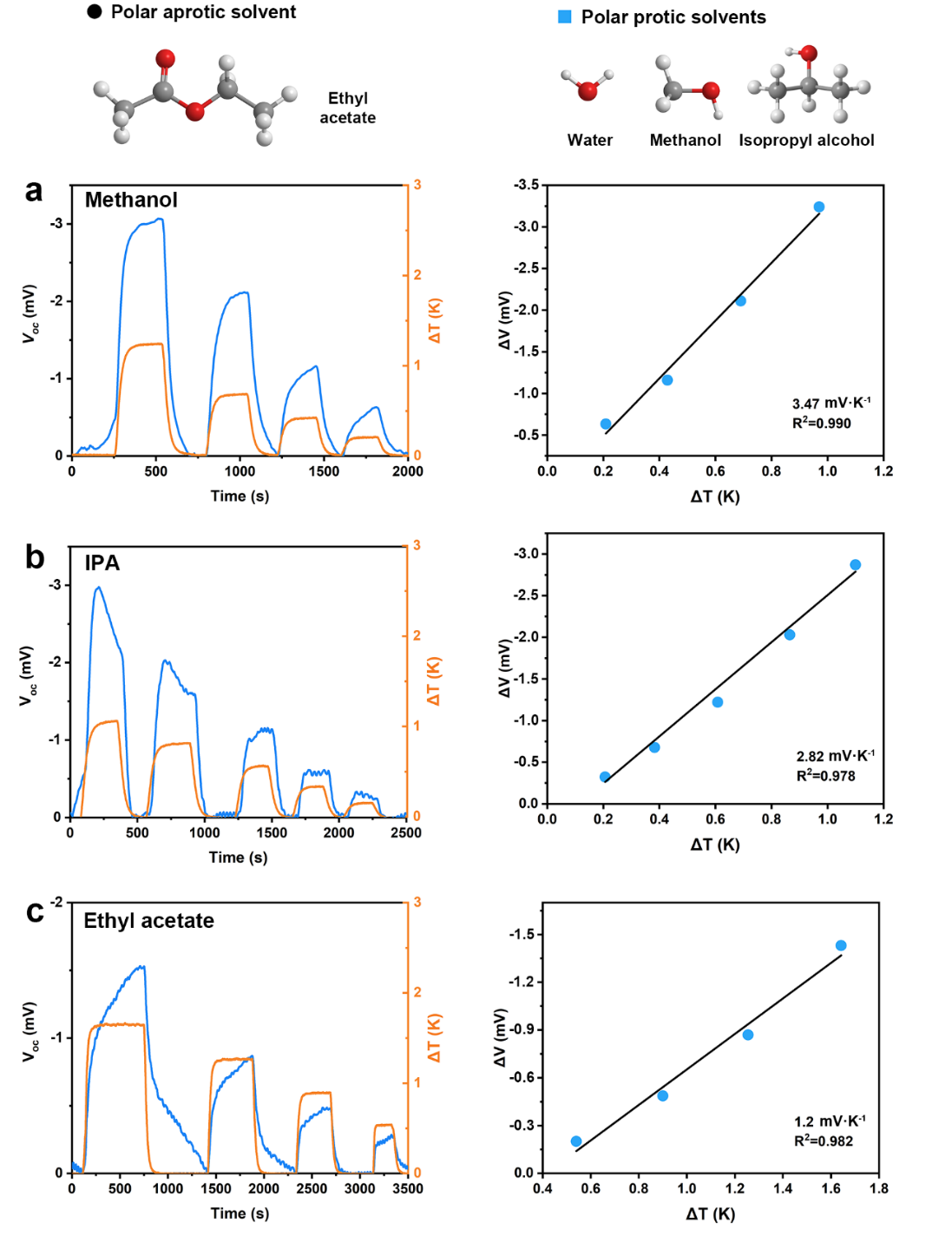


Figure S13. Schematic illustration of aprotic and protic solvents, along with *V*_oc_ and Δ*T* profiles of PAETC hydrogels with water partially substituted by (a) methanol, (b) IPA, and (c) ethyl acetate. Thermopower is extracted through linear fitting of the Δ*V*−Δ*T* data.

Figure S14. Water drip experiment at the hot and cold ends of the hydrogel. The promoting effect of water on proton dissociation was demonstrated using a simple drip experiment. Under a fixed temperature gradient, the addition of 1 μL DI water at the cold end significantly enhanced *V*_oc_ (Stage II). In contrast, introducing the same amount of DI water at the hot end initially reduced *V*_oc_ (Stage III). In Stage IV, after the temperature gradient was removed, protons redistributed to achieve a homogeneous distribution.


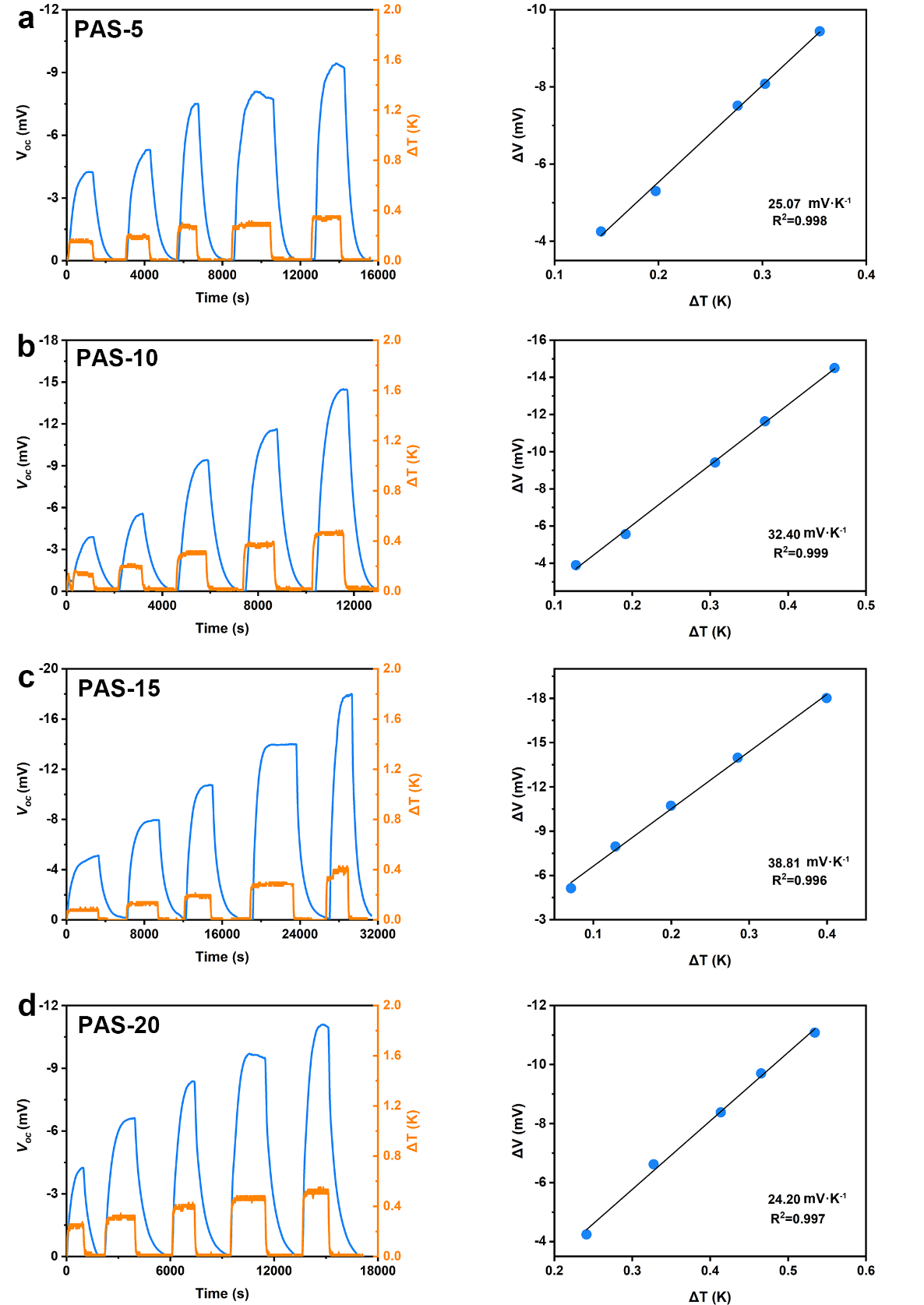


Figure S15. *V*_oc_ and Δ*T* profiles of PAETC/PSS (PAS) hydrogels under open conditions: (a) PAS-5, (b) PAS-10, (c) PAS-15, and (d) PAS-20. Thermopower is extracted through linear fitting of the Δ*V*−Δ*T* data.


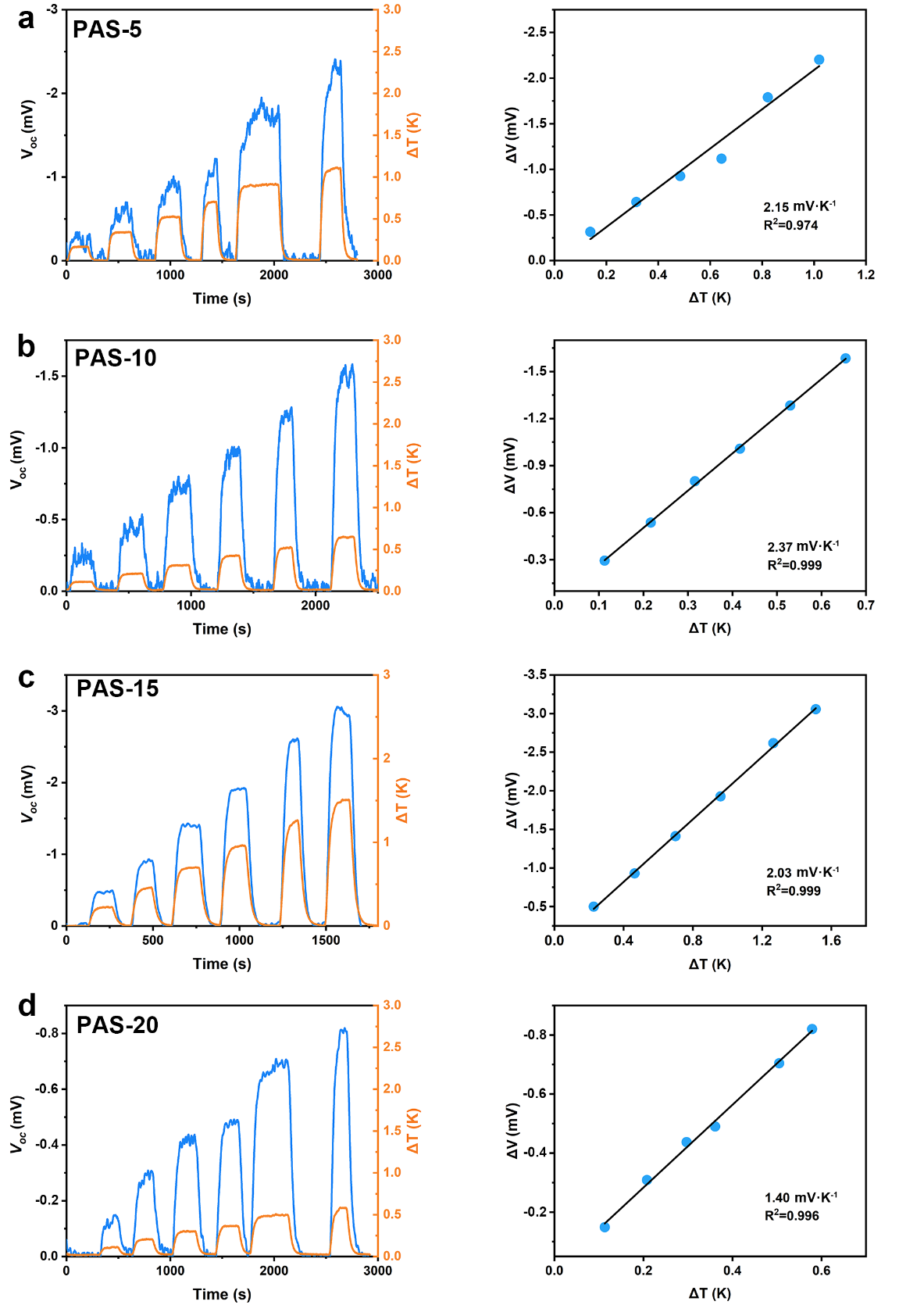


Figure S16. *V*_oc_ and Δ*T* profiles of PAS hydrogels under sealed conditions: (a) PAS-5, (b) PAS-10, (c) PAS-15, and (d) PAS-20. Thermopower is extracted through linear fitting of the Δ*V*−Δ*T* data.


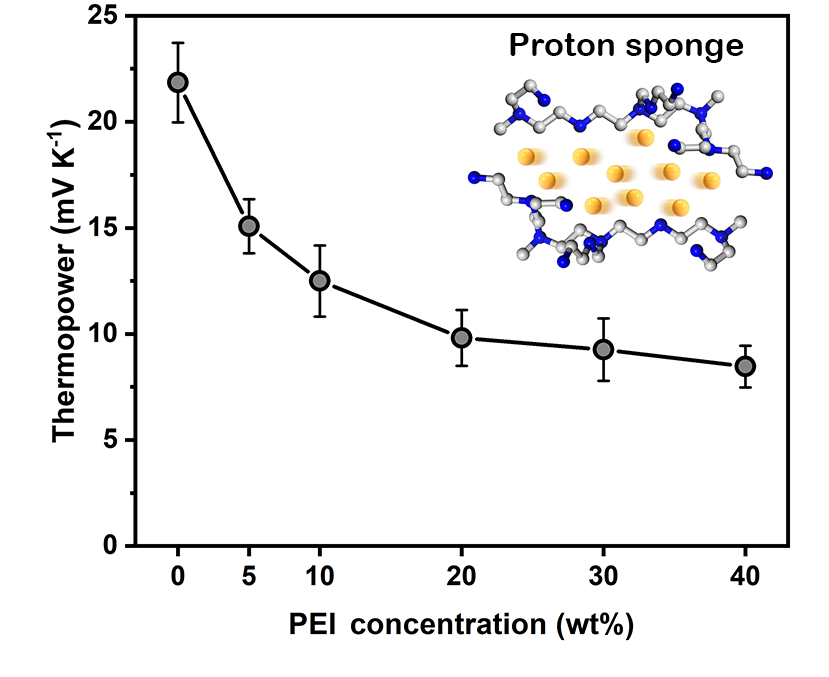


Figure S17. Thermopower of PAETC/PEI hydrogels.


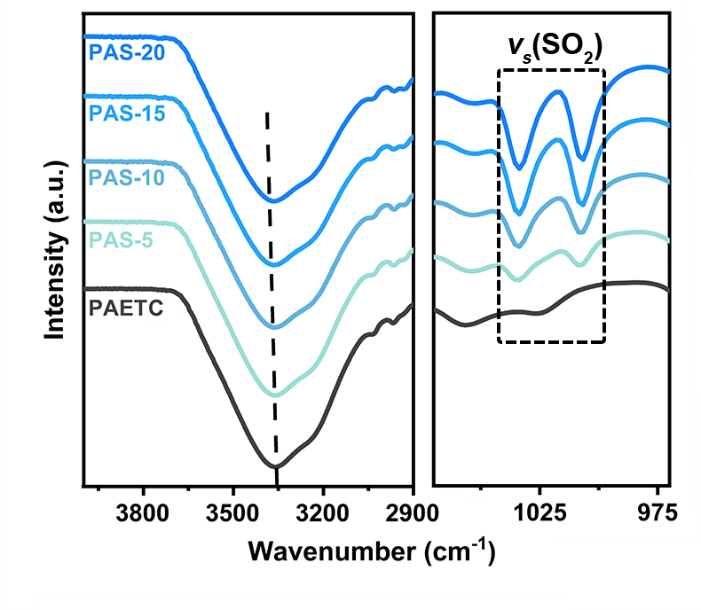


Figure S18. ATR-FTIR spectra of PAETC and PAS hydrogels highlighting the characteristic sulfonic acid vibrations.

**b**

**a**

Figure S19. (a) XPS O 1s and (b) N 1s spectra of PAETC and PAS-15 hydrogels.


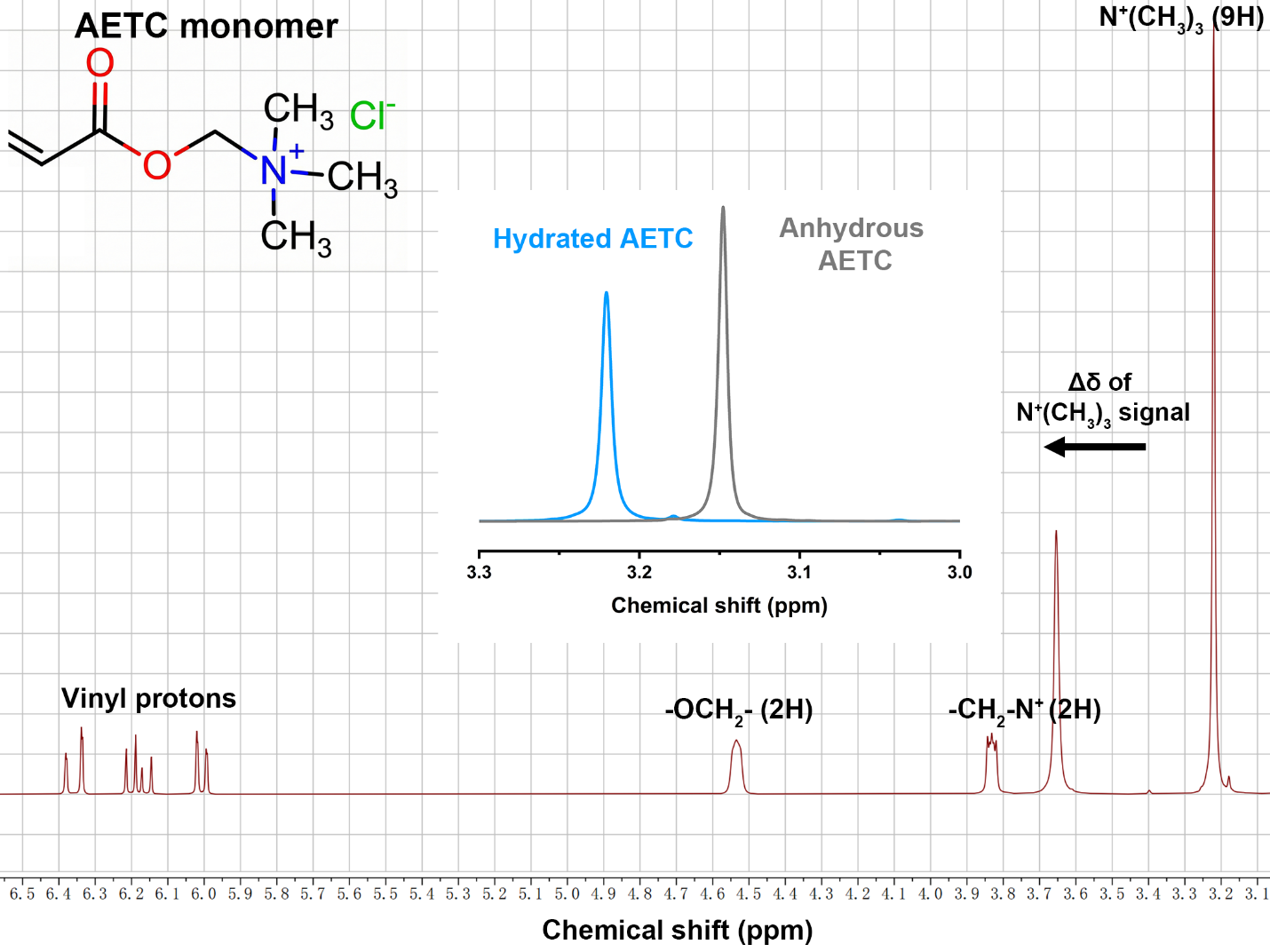


**Figure S20.** ^1^H NMR spectrum of AETC in DMSO-d_6_. The inset shows the enlarged view of N^+^(CH_3_)_3_ region.

Figure S21. MSD curves of PAS-5, PAS-10, PAS-15, PAS-20, and PSS. In stage I, under conditions of high polymer crowding, nanoclustered water confined within nanometer-sized cavities predominantly governs H_3_O^+^ diffusion. In stage II, H_3_O^+^ ions enter a diffusive regime within the polymer network, as indicated by the MSD exhibiting linear dependence on time (*t*). According to Einstein’s relation, the effective proton diffusion coefficient can be calculated from the slope of the MSD in stage II.


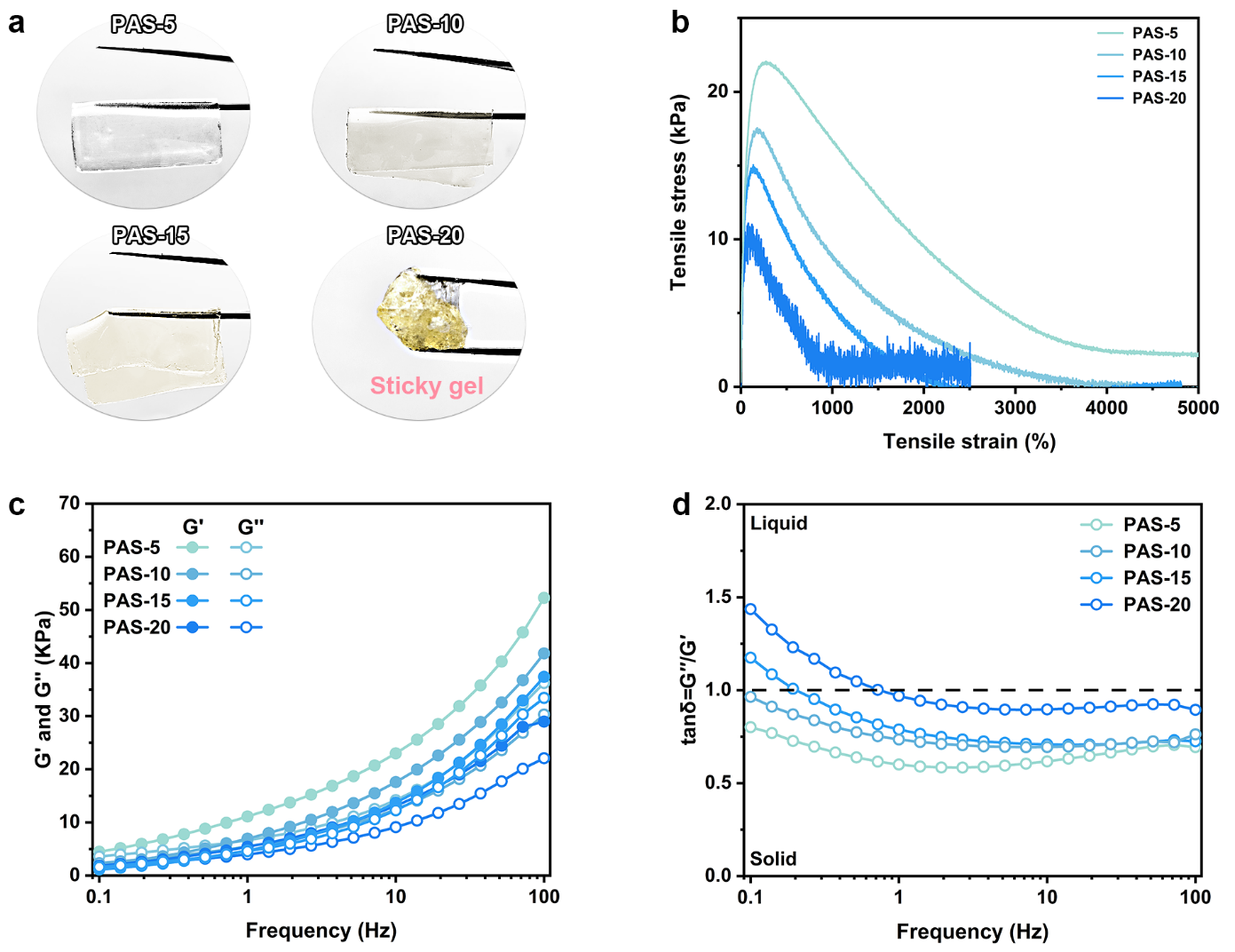


Figure S22. (a) Photographs of PAS hydrogels with varying PSS contents. (b) Tensile stress-strain curves of PAS hydrogels. Rheological properties of PAS-5, PAS-10, PAS-15, and PAS-20 hydrogels: (c) storage (*G*′) and loss (*G*″) moduli. (d) tan δ.


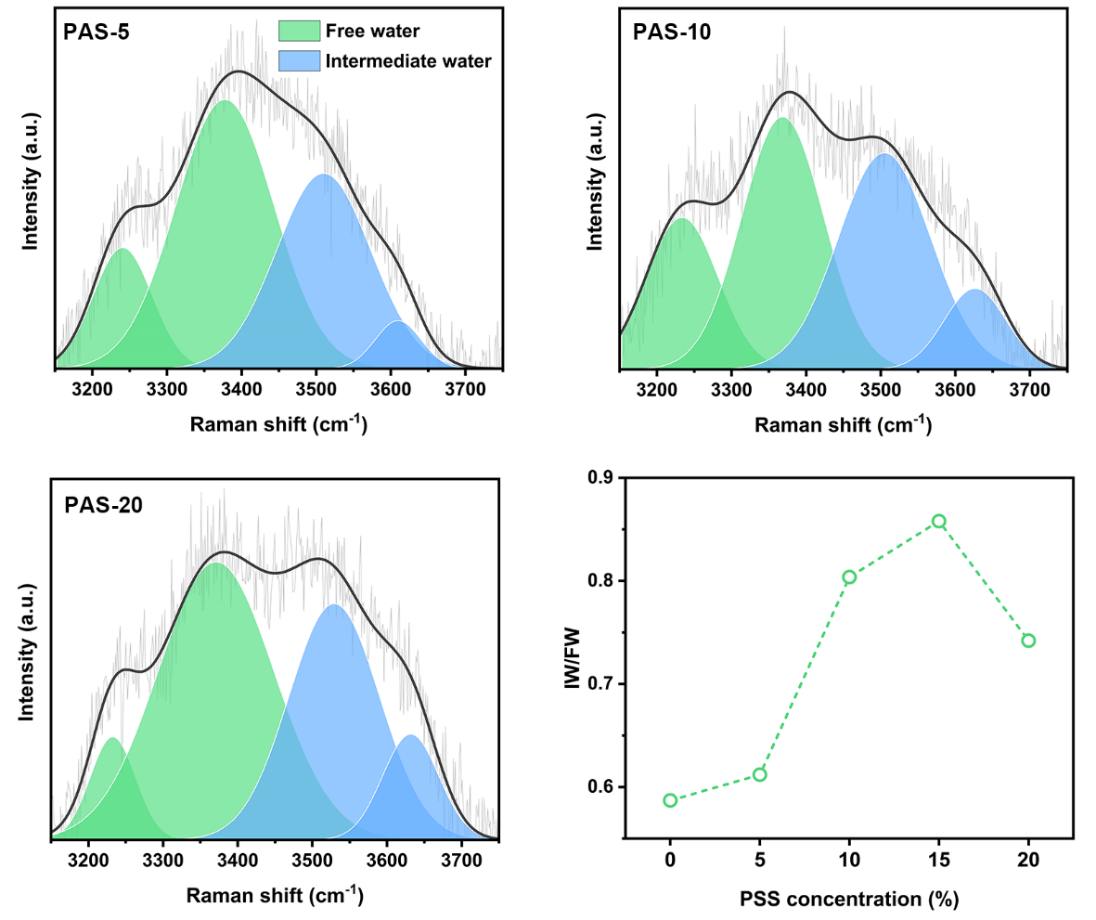


Figure S23. Deconvoluted Raman spectra of PAS-5, PAS-10, and PAS-20 hydrogels, along with the calculated IW/FW ratio at different PSS concentrations.


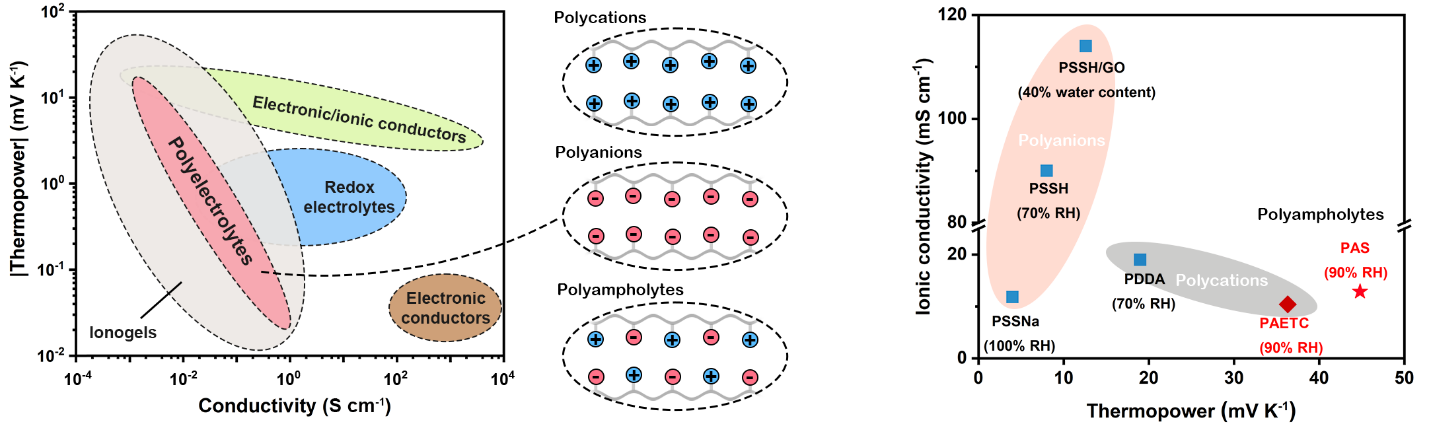


Figure S24. Comparison of thermopower and ionic conductivity of polyelectrolyte-based ionic thermoelectric (iTE) materials.


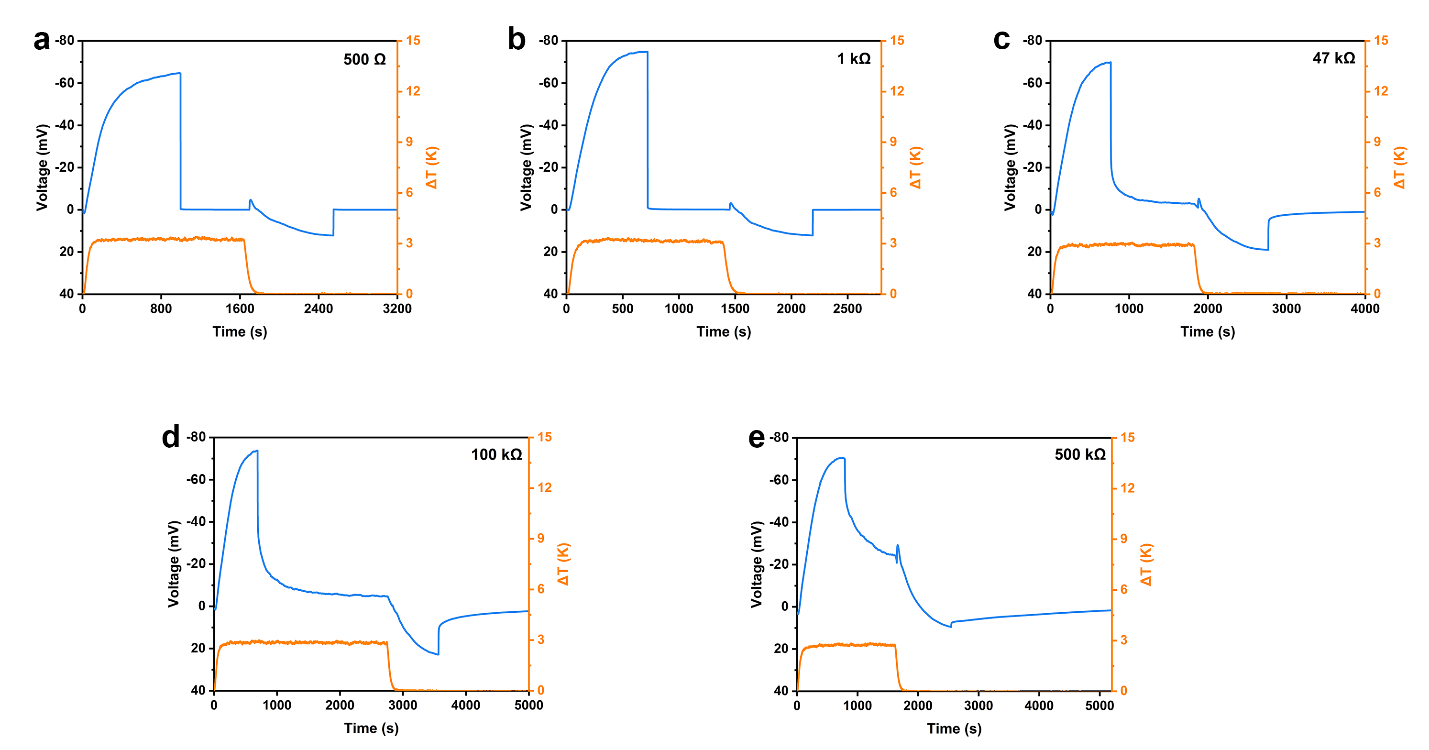


Figure S25. Voltage and Δ*T* profiles of an ionic thermoelectric capacitor (ITEC) for different external loads: (a) 500 Ω, (b) 1 kΩ, (c) 47 kΩ, (d) 100 kΩ, and (e) 500 kΩ.


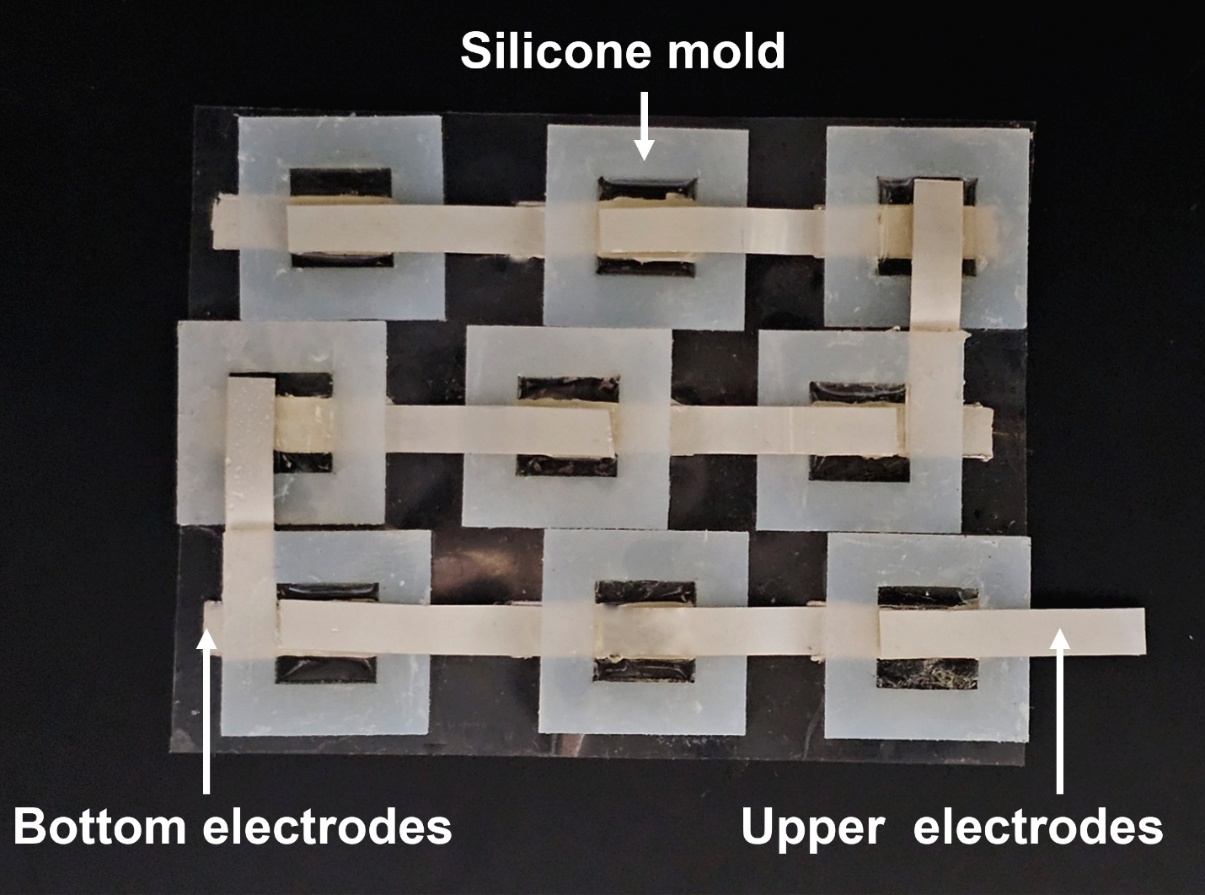


Figure S26. Photograph of the fabricated energy harvesting device.


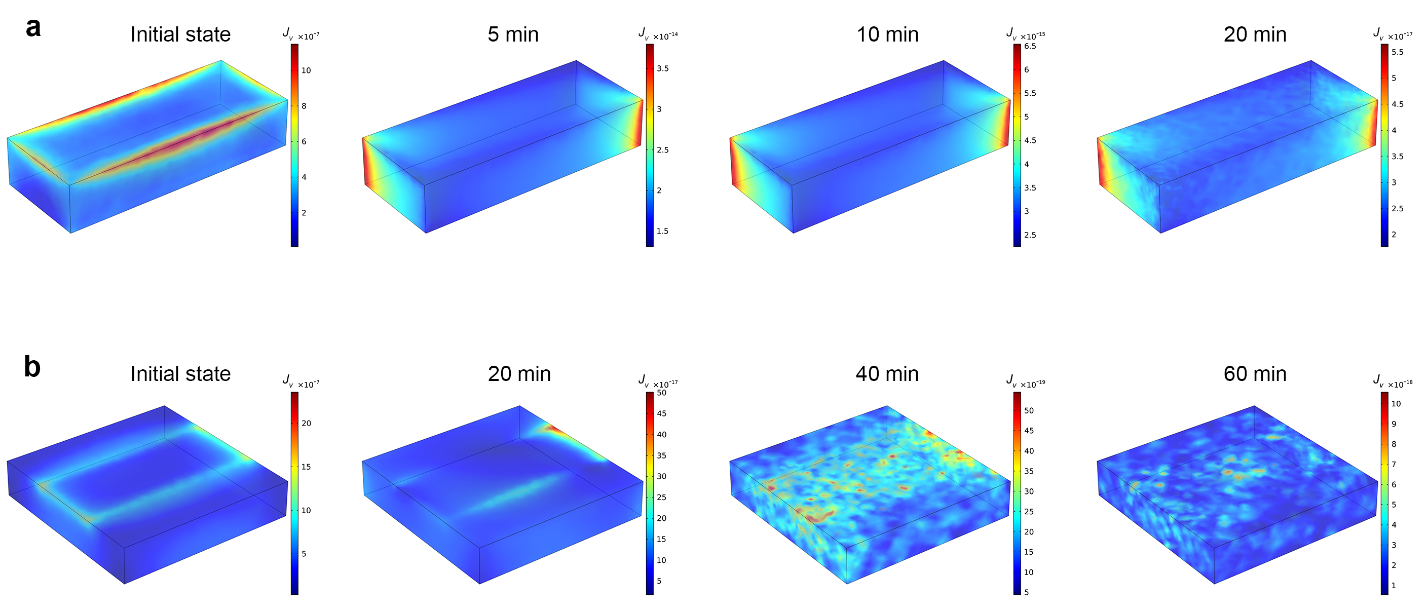


**Figure S27.**  Simulated total water vapor flux of the hydrogel under radiative heating from a 40 ℃ source: (a) sidewalls exposed to ambient, and (b) sidewalls and top exposed to ambient.


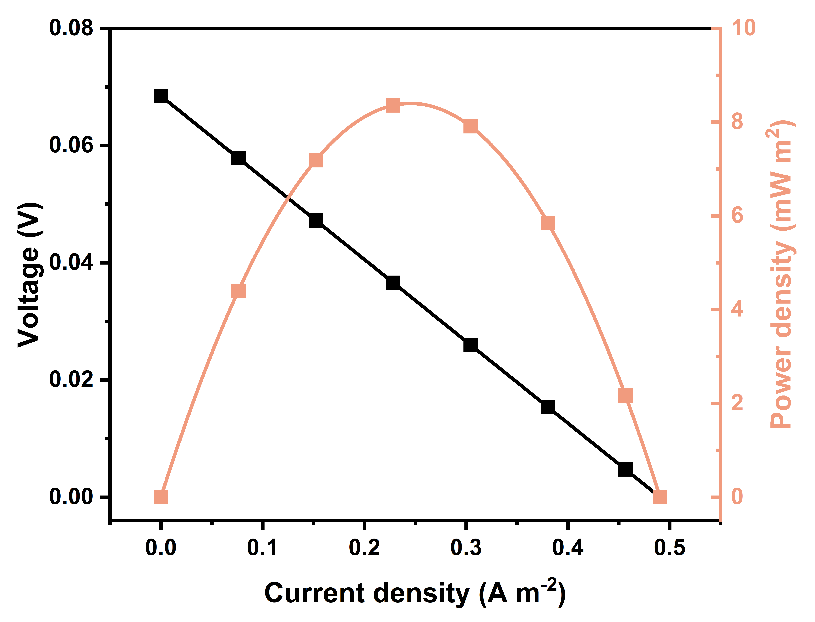


**Figure S28.** Voltage and power density versus current density curves for a single iTE element.


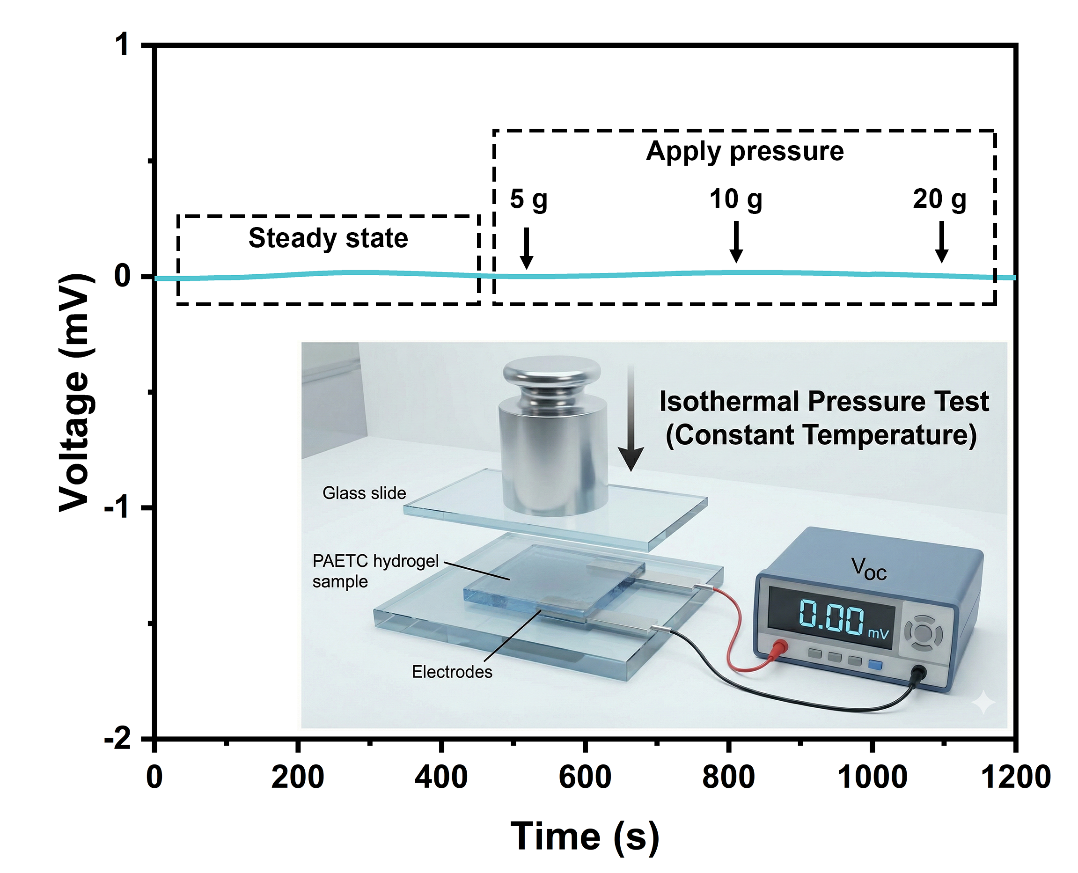


**Figure S29.** Isothermal pressure response of the hydrogel device for decoupling thermal and pressure signals. The temporal *V*_oc_ response under constant temperature shows a stable baseline and negligible voltage change upon sequential loading with 5, 10, and 20 g weights. The inset shows a schematic of the isothermal pressure-testing setup, in which the hydrogel sample is sandwiched between two glass slides and subjected to stepwise normal loading while *V*_oc_ is monitored.


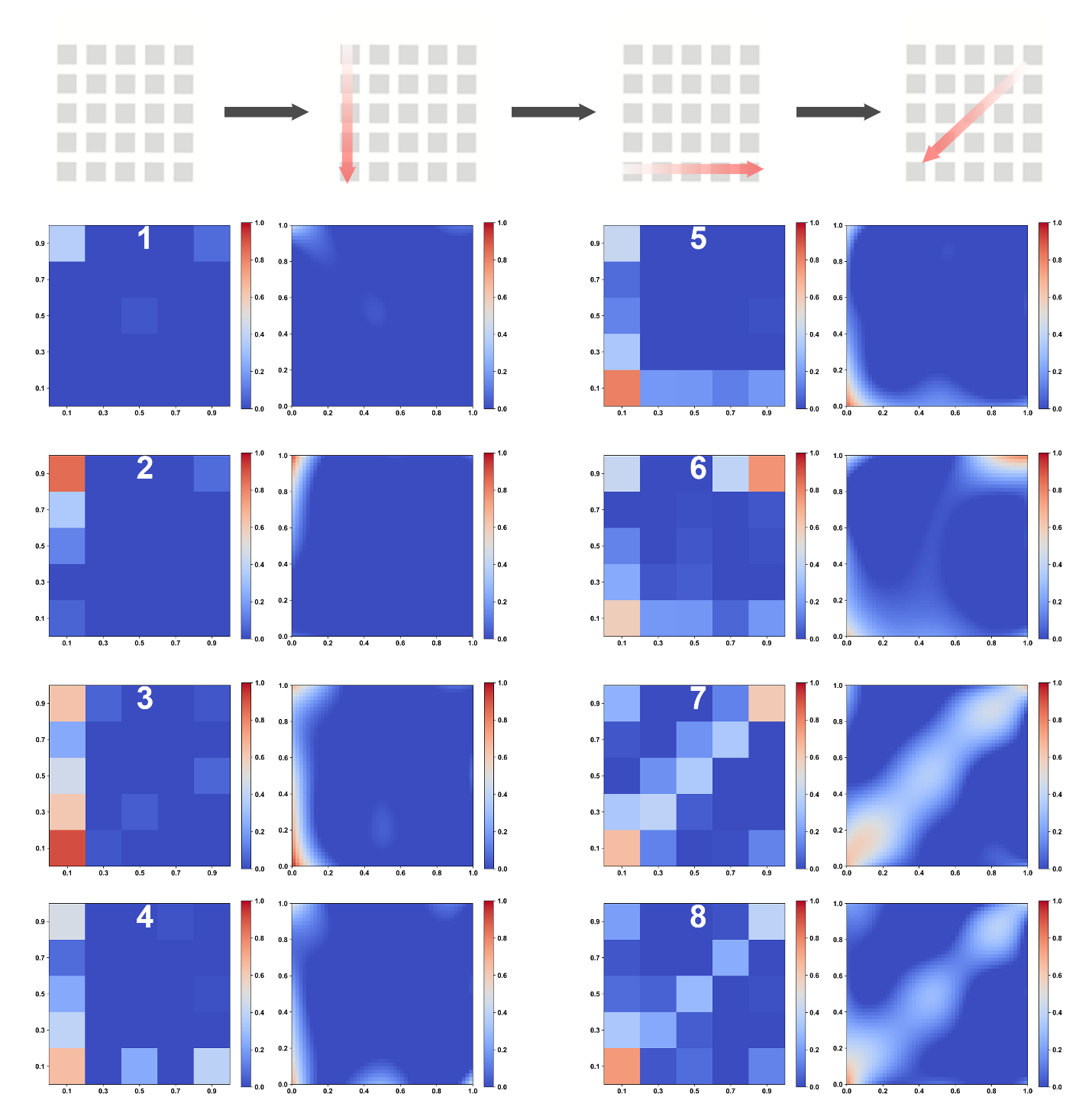


**Figure S30.** Resulting voltage mapping of the array sensing the direction and temperature of the heat trace.


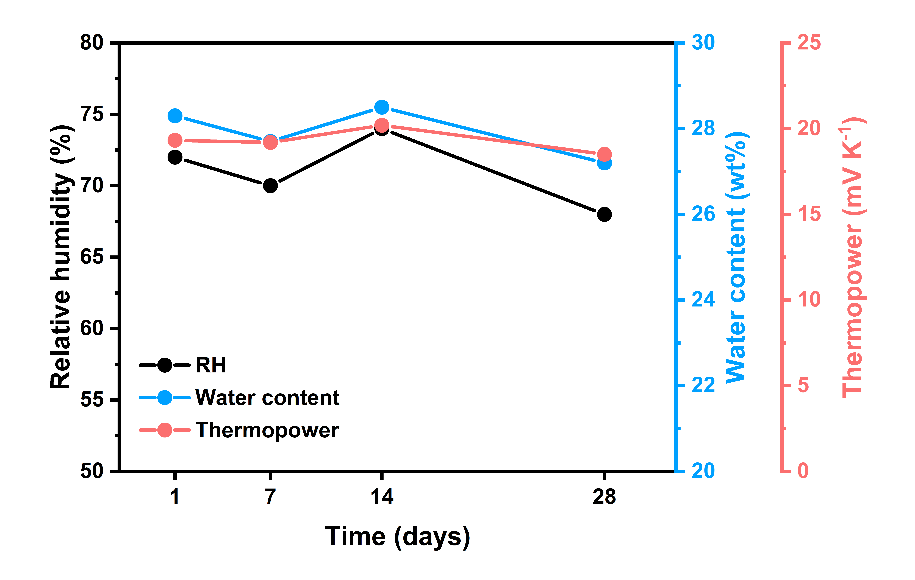


**Figure S31.** Long-term stability of the PAETC hydrogel under open environmental conditions.

The long-term stability of the PAETC hydrogel under open environmental conditions was evaluated over one month. Samples were stored in ambient air (20-25 °C, 60-80% RH) without encapsulation. Periodic measurements showed that variations in water content and thermopower closely tracked changes in ambient humidity, indicating that the device response is primarily governed by environmental moisture rather than intrinsic degradation of the hydrogel material.

Figure S32. Performance metrics comparison of the PAETC-based thermal sensor in this work with other reported ionic thermal sensors.

**Table S1.** Comparison of the PAETC-based thermal sensor in this work with other reported ionic thermal sensors.

| Materials | Sensitivity  (mV K⁻^1^) | Response time (s) | Flexibility | Stability | Application | Ref. |
| --- | --- | --- | --- | --- | --- | --- |
| PVDF-HFP/Na[TFSI]/ propylene carbonate | ~20 | / | Yes | / | Light-induced sensors | 1 |
| Polyethylene glycol-NaOH/LiI-MI_2_ | 10.6 | / | No | / | Liquid thermocouple | 2 |
| Paper/[EMIM][AC] | 2.2 | / | Yes | / | Fire alarming | 3 |
| Cement/carbon fiber/fly ash | 3.98 | / | No | / | Intelligent infrastructures | 4 |
| PVDF-HFP/C616 | 0.51 | / | Yes | / | Deep sea detection | 5 |
| Poly(AM-co-SBMA)/PDADMAC | 1.21 | / | Yes | / | Multisensory functions | 6 |
| PAM/K_4_Fe(CN)_6_/K_3_Fe(CN)_6_ | -1.21 | / | Yes | / | Ionic skins | 7 |
| PAA-MXene | 2.5 | 1.5 | Yes | / | Energy‑autonomous  motion sensors | 8 |
| Bacterial cellulose Fe(CN)_6_^3- /4-^ | -4.5  0.72 | / | Yes | / | Temperature sensor array | 9 |
| PAETC | 2.0 | 0.5 | Yes | > 90 days | Thermal sensor array | This work |

**References**

1. C. Chi, M. An, X. Qi, Y. Li, R. Zhang, G. Liu, C. Lin, H. Huang, H. Dang, B. Demir, Y. Wang, W. Ma, B. Huang, X. Zhang, Selectively tuning ionic thermopower in all-solid-state flexible polymer composites for thermal sensing. Nature Communications 13, 221 (2022).

2. N. Inomata, N. V. Toan, T. Ono, Liquid Thermocouple Using Thermoelectric Ionic Liquids. IEEE Sensors Letters 3, 1-4 (2019).

3. X. Wu, N. Gao, X. Zheng, X. Tao, Y. He, Z. Liu, Y. Wang, Self-Powered and Green Ionic-Type Thermoelectric Paper Chips for Early Fire Alarming. ACS Applied Materials & Interfaces 12, 27691-27699 (2020).

4. Y. Wei, Y. Cui, Y. Wang, Ionic thermoelectric effect of pure cement paste and its temperature sensing performance. Construction and Building Materials 364, 129898 (2023).

5. Y. Zhang, D. Ye, M. Li, X. Zhang, C.-a. Di, C. Wang, Solid state ionics enabled ultra-sensitive detection of thermal trace with 0.001K resolution in deep sea. Nature Communications 14, 170 (2023).

6. X. Fu, Z. Zhuang, Y. Zhao, B. Liu, Y. Liao, Z. Yu, P. Yang, K. Liu, Stretchable and Self-Powered Temperature–Pressure Dual Sensing Ionic Skins Based on Thermogalvanic Hydrogels. ACS Applied Materials & Interfaces 14, 44792-44798 (2022).

7. X. Lu, D. Xie, K. Zhu, S. Wei, Z. Mo, C. Du, L. Liang, G. Chen, Z. Liu, Swift assembly of adaptive thermocell arrays for device-level healable and energy-autonomous motion Sensors. Nano-Micro Letters 15, 196 (2023).

8. Y. Zong, H. Li, X. Li, J. Lou, Q. Ding, Z. Liu, Y. Jiang, W. Han, Bacterial cellulose-based hydrogel thermocells for low-grade heat harvesting. Chemical Engineering Journal 433, 134550 (2022).

9. X. Liu, X. Ji, R. Zhu, J. Gu, J. Liang, A Microphase‐Separated Design toward an All‐Round Ionic Hydrogel with Discriminable and Anti‐Disturbance Multisensory Functions. Advanced Materials 36, 2309508 (2024).
